# Classical singers are also proficient in non-classical singing

**DOI:** 10.3389/fpsyg.2023.1215370

**Published:** 2023-10-25

**Authors:** Camila Bruder, Pauline Larrouy-Maestri

**Affiliations:** ^1^Department of Music, Max Planck Institute for Empirical Aesthetics, Frankfurt am Main, Germany; ^2^Max Planck-NYU Center for Language, Music, and Emotion (CLaME), New York, NY, United States

**Keywords:** music, performance, acoustics, versatility, singing styles

## Abstract

Classical singers train intensively for many years to achieve a high level of vocal control and specific sound characteristics. However, the actual span of singers’ activities often includes venues other than opera halls and requires performing in styles outside their strict training (e.g., singing pop songs at weddings). We examine classical singers’ ability to adjust their vocal productions to other styles, in relation with their formal training. Twenty-two highly trained female classical singers (aged from 22 to 45 years old; vocal training ranging from 4.5 to 27 years) performed six different melody excerpts a cappella in contrasting ways: as an opera aria, as a pop song and as a lullaby. All melodies were sung both with lyrics and with a /lu/ sound. All productions were acoustically analyzed in terms of seven common acoustic descriptors of voice/singing performances and perceptually evaluated by a total of 50 lay listeners (aged from 21 to 73 years old) who were asked to identify the intended singing style in a forced-choice lab experiment. Acoustic analyses of the 792 performances suggest distinct acoustic profiles, implying that singers were able to produce contrasting sounding performances. Furthermore, the high overall style recognition rate (78.5% Correct Responses, hence CR) confirmed singers’ proficiency in performing in operatic style (86% CR) and their versatility when it comes to lullaby (80% CR) and pop performances (69% CR), albeit with occasional confusion between the latter two. Interestingly, different levels of competence among singers appeared, with versatility (as estimated based on correct recognition in pop/lullaby styles) ranging from 62 to 83% depending on the singer. Importantly, this variability was not linked to formal training *per se*. Our results indicate that classical singers are versatile, and prompt the need for further investigations to clarify the role of singers’ broader professional and personal experiences in the development of this valuable ability.

## 1. Introduction

Classical singers invest years in training to acquire and master a very specific technique. However, the reality of their professional lives often leads them to look for opportunities outside their strict field of training – for instance, performing at weddings or other social events, taking requests to sing pop songs, or even crossing over to musical theater, often without ever properly learning a technique to perform contemporary commercial music (CCM). LeBorgne and Rosenberg (2021) refer to the “hybrid singer” as a highly skilled vocal athlete, able to perform in multiple vocal styles, possessing a solid vocal technique that is “responsive, adaptable, and agile in order to meet demands of current and ever-evolving vocal music industry genres” (p. XV). Nevertheless, they note that the assumption that traditional classical pedagogy can support any style of singing is inconsistent with scientific findings about physiologic differences between classical and CCM styles of singing. Indeed, the growing number of books (Spivey et al., 2018; LeBorgne and Rosenberg, 2021) and dissertations (e.g., Hall, 2006; Willis-Lynam, 2015; Wilson, 2019) about how to teach classical singers to also (healthily) perform musical theater indicates the high demand for singers to (learn to) be versatile. In this study, we investigate the versatility of a cohort of classical singers by examining the acoustic characteristics of singers’ performances in contrasting styles, as well as the perception of these performances by lay listeners. We also explore the relationship between singers’ versatility and their music training.

According to Edith Bers, Chair of the Julliard Voice Department, it takes about ten years for a classical singer to be ready to begin a career (Kennedy Center, n.d.). Over the years, singers learn to master the mechanisms of vocal production. Following the source-filter theory of voice production (Fant, 1960), the acoustical properties of the voice result from the combination of voice source and vocal tract resonances. Concretely, the subglottal pressure, as well as the tensing and stretching of the vocal folds, and the glottal adduction, collectively modulate the frequency of the airflow going through the vocal folds, which in turn determines the fundamental frequency (*𝑓ₒ*). This airflow is then filtered by the vocal tract, which selectively enhances the amplitude of certain partials of the voice source spectrum, via changes in the position of articulators (lips, tongue, lower jaw, velum, pharyngeal walls, and larynx). These alterations in the configuration of the vocal tract lead to formants, that is, bands of enhanced power in the resulting sound (ANSI, 2004; Titze et al., 2015). Specifically, changes in the vocal tract resonance frequencies *𝑓*_R1_ and *𝑓*_R2_ (and resulting formants F1 and F2) play a central role in determining vowel quality. Beyond being involved in speech production, this complex machinery allows for the specific acoustic characteristics of classical singing, as summarized by Sundberg (2013). One example is the so-called singer’s formant cluster, most clearly described for male voices. Trained male classical singers can produce voice spectra in which the partials falling in the frequency region around 2,5–3 kHz are greatly enhanced, leading to a peak in the spectral envelope. This phenomenon is explained as the acoustic consequence of clustering of the resonances *𝑓*_R3_, *𝑓*_R4_ and *𝑓*_R5_ (Sundberg, 1974). Another example, mostly concerning female singers, is the resonance tuning strategy in high-pitched singing, which consists of widening the jaw opening so that *𝑓*_R1_ is shifted to a frequency near *𝑓ₒ*, resulting in considerable gain in amplitude of a specific frequency zone (Sundberg, 1975; Joliveau et al., 2004; Garnier et al., 2010). Importantly, both the resonance tuning strategy in high-pitched singing and the singer’s formant cluster are resonatory phenomena that increase singers’ audibility in the presence of orchestral accompaniment without additional vocal effort (i.e., they allow for vocal economy). This contrasts with contexts like pop singing, where singers typically use a microphone, or lullaby singing, where the intimate setting of close proximity to an infant requires very soft singing. Also noteworthy are nonlinear source-filter interactions, which may make vocal fold vibration unstable when *𝑓ₒ* approaches *𝑓_R1_* (Titze, 2008; Titze et al., 2008; Kaburagi et al., 2019). These interactions are especially relevant and frequent for female high-pitched singers, who must skillfully mitigate them to avoid qualitative changes in timbre and volume.

Another important feature of classical singing is the extensive use of vibrato, that is, a periodic oscillation in the *𝑓ₒ* that develops automatically with training (Bjørklund, 1961; Sundberg, 1994). Voice pedagogues tend to agree that a healthy, well-trained voice will naturally have vibrato (e.g., Miller, 1986). According to Sundberg (2013), the use of vocal vibrato may eliminate beats with the sound of a vibrato-free accompaniment, providing classical singers with some freedom in intonation and allowing thus for greater emotional expression in singing. Likewise, the ability to sing with a “straight tone” – i.e., healthy, unconstrained singing, that is perceived as singing without vibrato, even though there might be oscillations in the acoustic signal – may be seen as a sign of versatility, showing that singers can skillfully control their vibrato to fit any style (Katok, 2021).

In addition to having a wide vibrato, the operatic singing style has been described as more irregular and chaotic than other styles: based on archetypical singing voice samples from different styles (opera, country, soul, jazz, musical theater, and pop), Butte et al. (2009) used nonlinear dynamic analysis of the correlational dimension (the D_2_ measure), as well as usual voice perturbation measures, to compare styles. They found higher shimmer and D_2_ values for operatic than other singing styles, as well as higher jitter for operatic, pop and soul than other styles. Similarly, Larrouy-Maestri et al. (2014) compared singing performances of the same melodies with and without use of the classical singing technique and described higher jitter and shimmer and lower signal-to-noise ratio (as well as wider vibrato extent and slower tempo) in operatic singing, supporting the (somewhat counterintuitive) idea that the waveform resulting from classical singing is more irregular than that from other singing styles.

A different approach to describe different singing styles focuses on production mechanisms and the voice source. Thalen and Sundberg (2001) recorded performances by one singer proficient in classical, pop, jazz and blues styles, and analyzed perceived phonatory pressedness in relation to markers of vocal production function (inverse filtering and glottogram data). They proposed characterizing singing styles based on modes of phonation (which are related to different degrees of airflow and vocal fold adduction force: breathy, flow, neutral and pressed phonation modes of phonation have been described – Sundberg, 1987). Thalen and Sundberg (2001) suggested that classical singing is usually close to flow phonation, pop and jazz singing have values closer to flow than pressed phonation, and blues singing lies close to pressed phonation. For comparison, the pop style is typically represented by performers like Randy Crawford and Whitney Houston; the jazz style, by performers like Billie Holiday and Sarah Vaughan; and the blues style by performers like Bessie Smith and Janis Joplin (examples given by Sundberg et al., 2004).

Singing expertise demands highly developed motor control, which relies on auditory and kinesthetic feedback (Wyke, 1974). Both aural and kinesthetic awareness are thus encouraged by voice pedagogues (e.g., Ohrenstein, 2003). Such training leads to a particular role of kinesthetic control in classical singers compared both to non-singer musicians and to non-musicians, as indicated by the effect of masking noise in intonation accuracy (Mürbe et al., 2004; Jones and Keough, 2008; Erdemir and Rieser, 2016). This ability is also demonstrated in brain imaging studies showing that classical singing expertise coincides with the development of enhanced somatosensory processing, representing proprioceptive feedback from the articulators and the larynx (Kleber et al., 2010). Classical singing expertise is also related to increased involvement of the cerebellum and implicit motor memory areas at the subcortical level, and to a fronto-parietal network associated with action monitoring and sensory guidance of motor activity (Kleber et al., 2010).

While classical singing training results in specific acoustic patterns and systematic bodily changes, it is not clear how it affects a singer’s ability to produce diverse sound qualities when singing in other styles. That is to say, to convincingly perform in other styles, singers may need to suppress or adapt muscular programs acquired during their intense training. To the best of our knowledge, empirical investigations about the proficiency of classical singers in other styles have not yet been conducted.

Here, we examine this ability by focusing on a cohort of classical singers performing the same melodies in three contrasting ways: singers were instructed to sing as if they wanted to make a baby sleep; as if they were singing a pop song with a microphone; and as if they were singing an opera aria on stage. We use the term “style” operationally, with the meaning of contrasting functions and resulting sound qualities. For pragmatic reasons, we chose styles that classical singers could perform without having to learn a further specific singing technique (such as belting). Since we did not provide singers with any definition of “pop,” and given the broad use of this term, the pop singing we report here is directly related to the stylistic conceptions and abilities of our particular cohort of (Brazilian) classical singers. Regarding the lullaby singing, we refer to the typical singing used to soothe an infant. Lullabies are usually simple, repetitive melodies, with simple rhythm and a preponderance of small melodic steps, and are typically performed a cappella, with soft and quiet singing by a caregiver (Unyk et al., 1992; Trehub and Trainor, 1998; Mehr et al., 2019). Such typical features allow lullabies to be cross-culturally recognized when compared to matched adult-directed songs (Trehub et al., 1993) or as “music to soothe an infant” (relative to dance, healing or love uses of songs) (Mehr et al., 2018; Yurdum et al., 2023).

Apart from investigating and comparing acoustic characteristics of contrasting singing performances, we also examine singers’ versatility through the listener perspective, in a lab experiment where lay listeners performed a style recognition task.

## 2. Part I: acoustic characteristics of singing performances

### 2.1. Method

#### 2.1.1. Singer participants

Twenty-two highly trained Brazilian female classical singers (16 sopranos, 6 mezzo-sopranos, aged from 22 to 51 years old, *M* = 32.5, *SD* = 7.1) were recruited *via* personal contact. They had between 4.5 and 27 years of training in classical singing (*M* = 12.9 years, *SD* = 6). All of them also declared having experience performing in other styles: 14 in pop, 13 in MPB (Música Popular Brasileira, a genre of popular Brazilian Music), five in jazz, three in gospel, one in musical theater (multiple responses possible for each singer). They reported spending between one and 40 h per week performing (*M* = 15.9 h, *SD* = 9.9) at the time of the recording (including the time spent practicing). Five singers reported singing exclusively as soloists, five indicated singing about 75% of the time as soloists (and 25% of the time in a choir), five indicated singing about half the time as soloists, and seven indicated singing about 25% of the time as a soloist (and 75% in a choir). Singers also reported having started voice lessons between ages of six and 25 years old (*M* = 17.7 years, *SD* = 5.7), having between four and 30 years of music training (*M* = 15.7, *SD* = 7.3) and playing an instrument between zero and 15 years (*M* = 4.5 years, *SD* = 3.9). They also reported having had on average between zero and eight performances per month in the last 12 months (*M* = 2.9, *SD* = 2.4), including online versions of events due to the COVID-19 pandemic. Singers’ characteristics are summarized in Supplementary Table S1.^1^

#### 2.1.2. Material

The melody excerpts correspond to the first phrase of six different Brazilian songs: the lullabies Nana Nenê and Boi da Cara Preta; the play songs Alecrim and Nesta Rua (all very well known, traditional and anonymous Brazilian songs); the MPB song (Música Popular Brasileira, or popular Brazilian music) Chove Chuva by Brazilian artist Jorge Ben Jor (1939–); and the art song Melodia Sentimental, part of the symphonic poem A Floresta do Amazonas by Brazilian classical composer Heitor Villa-Lobos (1887–1959), with text by Dora Vasconcellos (1910–1973). Singers were provided with sheet music well in advance of their scheduled recording session to ensure thorough preparation. Most singers received sheet music between three and four weeks beforehand, together with their invitation to participate in the recording. However, four singers were brought in as last-minute substitutes due to others canceling participation, in which case they received sheet music at least two days before their recording session. The starting note of each melody was played on a keyboard by the researcher before each performance. Please see Supplementary Figure S1 for sheet music and Supporting Text 1 for translations of the texts from the melody excerpts. Performances in operatic singing were recorded with higher pitch than pop and lullaby performances, with the aim of producing naturalistic performances and keeping singers comfortable.^2^ This means that for all but one of the melodies, operatic singing was recorded one fifth higher than pop and lullaby singing. The exception was the melody Melodia Sentimental, which was recorded one fourth higher. This was done because of the melody’s extensive range, which would otherwise include a G5, potentially challenging for the mezzo-sopranos in the sample.

#### 2.1.3. Procedure

##### 2.1.3.1. Recordings

Singers were invited to a recording session of approximately one hour, in a professional music recording studio in São Paulo, Brazil, in March 2022. Recordings were made using an AKG C-414 B-ULS microphone in cardioid pattern, and using the Mac standard for lossless audio (Audio Interchange Format, AIFF), with 24 bits per sample and 44.1 kHz sampling rate. The recording procedure was consistent throughout all recording sessions. Singers were instructed to stand on fixed marks on the floor (though some movement during singing is unavoidable). The distance between singers’ mouths and the microphone was set to around 10 cm for the lullaby performances; around 30–40 cm for pop performances; and around 60 com for operatic performances.^3^ Singers were asked to perform each melody excerpt as a lullaby, as a pop song, and as an opera aria, and to additionally speak the corresponding lyrics as if speaking to an adult and as if speaking to an infant. Note that the speech performances are not analyzed in the current study.^4^ Singers received the following instructions:

- for lullaby singing: imagine you have a baby on your chest and you want to make it sleep;
- for pop singing: imagine you are performing a pop song using a microphone;
- for operatic singing: imagine you are on stage performing an opera aria.

Performances with a /lu/ sound were recorded directly after the corresponding performance with lyrics. The vowel /u/ was chosen based on the first author’s experience as a voice teacher and the observation that it is a comfortable vowel for Brazilian female singers to produce a homogenous sound. Each singer was thus recorded in 36 different singing conditions (six melodies, in three styles of singing and two types of production), for a total of 792 performances in this stimulus set (that is, resulting from 36 conditions performed by 22 singers).^5^ For each of the 36 conditions, at least three takes were recorded. At request of the recording technician and/or of the singers, one or two additional takes were occasionally recorded (in rare cases, between three and four extra takes were recorded for the same condition).

##### 2.1.3.2. Audio processing and take selection

Individual takes of recorded performances (lasting between 5 and 12 s) were cut using Audacity software (version 3.1.3). For each singer, one take for each of the 36 conditions was selected by the first author for further analysis, based on the following (admittedly arbitrary) criteria to exclude takes: (a) occasional ambient noise (e.g., coming from singers’ movements); (b) low vocal quality; (c) low expressiveness; (d) low authenticity.

##### 2.1.3.3. Loudness normalization

The final set of 792 stimuli was normalized to ensure a similar level of loudness within style, while keeping variability between styles (i.e., contrastive softness of lullabies compared to the higher intensity associated with operatic technique). Using the software To Audio Converter (version 1.0.16–1059), all opera stimuli were loudness normalized following the EBU R 128 standard (without any dynamic range compression) to −14 Loudness Units relative to Full Scale (LUFS); all pop singing stimuli to −18 LUFS; and all lullaby stimuli to −25 LUFS. Examples of the stimuli used in the present study are currently available at https://osf.io/6eyuc/.

#### 2.1.4. Acoustic analyses

Each of the 792 singing performances was segmented into individual notes using Tony (Mauch et al., 2015). After note corrections (made manually upon visual inspection of individual files), data about duration of each individual note were exported and used to extract individual notes of the melodies using a sox bash script. Consonants were kept at the beginning of each note. This procedure produced 9,108 chunks of individual notes. The average length of individual notes was 0.596 s (*SD* = 0.445, range: 0.081–3.240 s). For each note, we used Praat (Boersma, 2001; Version 6.0.46) with the settings pitch floor = 75 Hz and pitch ceiling = 800 Hz, to extract the measures: *𝑓ₒ*; *𝑓ₒ* max; *𝑓ₒ* min; standard deviation of the *𝑓ₒ*; shimmer_local (perturbation in the amplitude of *𝑓ₒ*); and jitter_local (perturbation in the periodicity of *𝑓ₒ*). Note that for jitter and shimmer, we observed measurement imprecision (aberrant values for very short notes), so we trimmed values higher than two standard deviations above the mean value before calculating average values per performance. As a consequence, we excluded 4% of individual note measurements for jitter and 2.6% for shimmer. Using VoiceSauce (Shue et al., 2011),^6^ with the same settings as in Praat mentioned earlier (and also based on individual notes), we also extracted the following measures – (a) Harmonics-to-noise ratio in the 0–3.5 kHz band (HNR35): the ratio between periodic and nonperiodic components of the signal, based on the algorithm described by Krom (1993). The HNR measurements are found by liftering the pitch component of the cepstrum and comparing the energy of the harmonics with the noise floor. (b) Cepstral peak prominence (CPP): a different voice quality measure of the relative levels of harmonic and inharmonic energy in the voice, based on the algorithm described by Hillenbrand et al. (1994). CPP is the dB difference between the cepstral peak and a linear regression line measured at the corresponding quefrency – where lower values have been perceptually associated to breathiness and dysphonia (Murton et al., 2020). (c) Energy (specifically, the Root Mean Square Energy): generally used to evaluate the amplitude of the audio signal.^7^ The extracted features were then averaged per take. We also computed pitch accuracy: we first converted *𝑓ₒ* values from Herz to cents (100 cents corresponds to one semitone, the reference lowest note used was 261.626 Hz), then calculated the absolute difference between these values and reference notes (i.e., “correct” notes, according to sheet music), also in cents; then averaged the pitch (in)accuracy per take. In addition, we used *𝑓ₒ* max-min as an approximation of vibrato extent based on Praat’s output of *𝑓ₒ* max and *𝑓ₒ* min (in Herz) of a selected long note from each performance (we used the same frequency whenever possible, whatever its position in the melodies; see the notes framed in blue in the respective sheet note in Supplementary Figure S1).

#### 2.1.5. Statistical analyses

All analyses were performed using R Statistical Software (version 4.1.2; R Core Team, 2021) and R Studio (version 2022.7.1.554; RStudio Team, 2022). To test whether acoustic features vary across styles, we ran a two-ways mixed design analysis of variance (ANOVA; with the aov function and default settings in R) for each acoustic feature, with factors Style (within participants) and Type of production (between participants). We also performed a principal component analysis (PCA, with the prcomp function in R) to explore the dimensionality of the acoustic space of the singing performances.

### 2.2. Results and discussion

**Figure 1:**
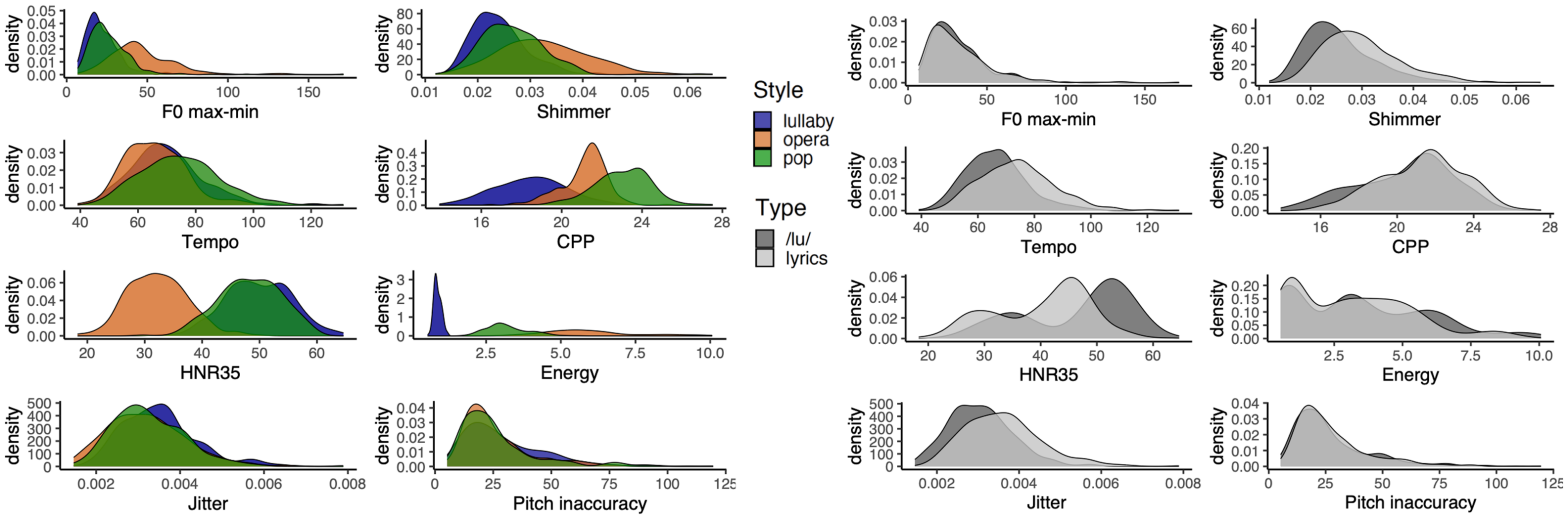
Density distribution of acoustic features by style (left) or type of performance (right).

**Figure 2:**
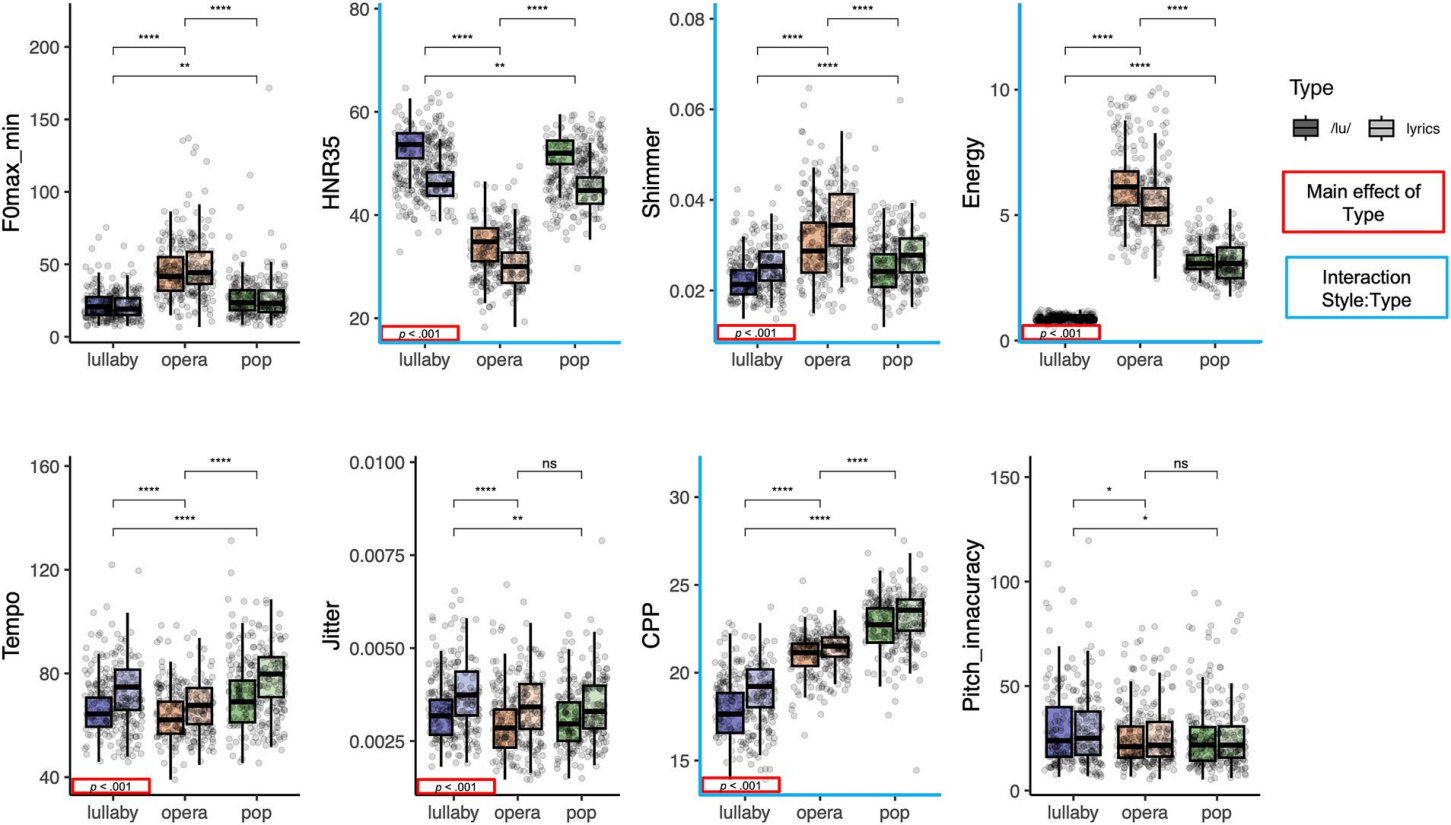
Boxplots displaying the distribution of the selected acoustic features for each of the three singing styles (lullaby, opera, pop), by type of production (with /lu/, illustrated with darker colors, and with lyrics, illustrated with lighter colors). Significance of the main effect Style of singing is depicted with stars and significance of the main effect Type of production is presented with red frames in the bottom-left corner of each plot. The blue axes indicate significant interaction between Style and Type of production. The Energy measure is depicted here for transparency (see Footnote 7).

A total of 792 performances, consisting of six melody excerpts performed by 22 singers, in three styles of singing and two types of production (with lyrics or a /lu/ sound) were analyzed. Each performance was around nine seconds long. The acoustic analysis reveals clear acoustic profiles for the different singing styles, supporting that singers’ productions are contrasted, as illustrated in Figures 1, 2. Please see Supplementary Tables S2, S3 for summary statistics of these measures, and Supplementary Figure S2 for a correlation matrix.

As shown in Figure 2, the ANOVAs showed differences between styles for all acoustic features (all *p*s < 0.001) and between types of performance for all features (all *p*s < 0.001), except for pitch accuracy and *𝑓ₒ* max-min. Also, there was a significant interaction between the main effects of style and type of performance in the case of shimmer, CPP, Energy and HNR35 (all *p*s < 0.05).

Comparing the acoustic profiles of the three styles of singing, we found that pop performances had faster tempo and higher CPP values than both other styles. The interpretation of CPP values for the singing voice is still unclear. Considering that Baker et al. (2022) report an interaction between *𝑓ₒ* and CPP levels, one could only directly compare CPP levels of pop and lullaby singing of our framework, in which case the lower values of CPP in lullaby singing seem to indicate a breathier voice quality (e.g., Murton et al., 2020). Lullabies also had smaller values of *𝑓ₒ* max-min (none or limited vibrato), higher jitter values, and lower pitch accuracy and shimmer. The higher values of jitter in lullabies may be linked to their soft phonation level: for spoken voices, a dramatic increase in jitter has been described below a critical threshold of 80 dB (Brockmann et al., 2008). The worse pitch accuracy might also be related to worse intonation control in soft phonation. Lullabies were also slower than pop performances. These features combined seem to represent the typical soft, slow and intimate singing used to soothe an infant. Turning to operatic performances, they were slower, had lower values of HNR35 and higher values of shimmer and *𝑓ₒ* max-min (indicating more extensive use of vibrato) than both other styles. This is in line with the general description of operatic singing by Larrouy-Maestri et al. (2014), with the exception that in that study the authors also reported higher jitter for performances in operatic (than non-operatic) style; and with the description of higher shimmer in operatic singing by Butte et al. (2009). The intermediary values of CPP for operatic singing (lower than in pop singing) are somewhat surprising: CPP values have been described to increase with sound pressure level and 𝑓ₒ (Brockmann-Bauser et al., 2021; Baker et al., 2022), so considering that operatic performances had higher pitch and sound pressure level than both other styles, it would make sense for them to also have higher CPP values. The use of vibrato may help explain this finding, but this is only speculatory at the moment.

**Figure 3:**
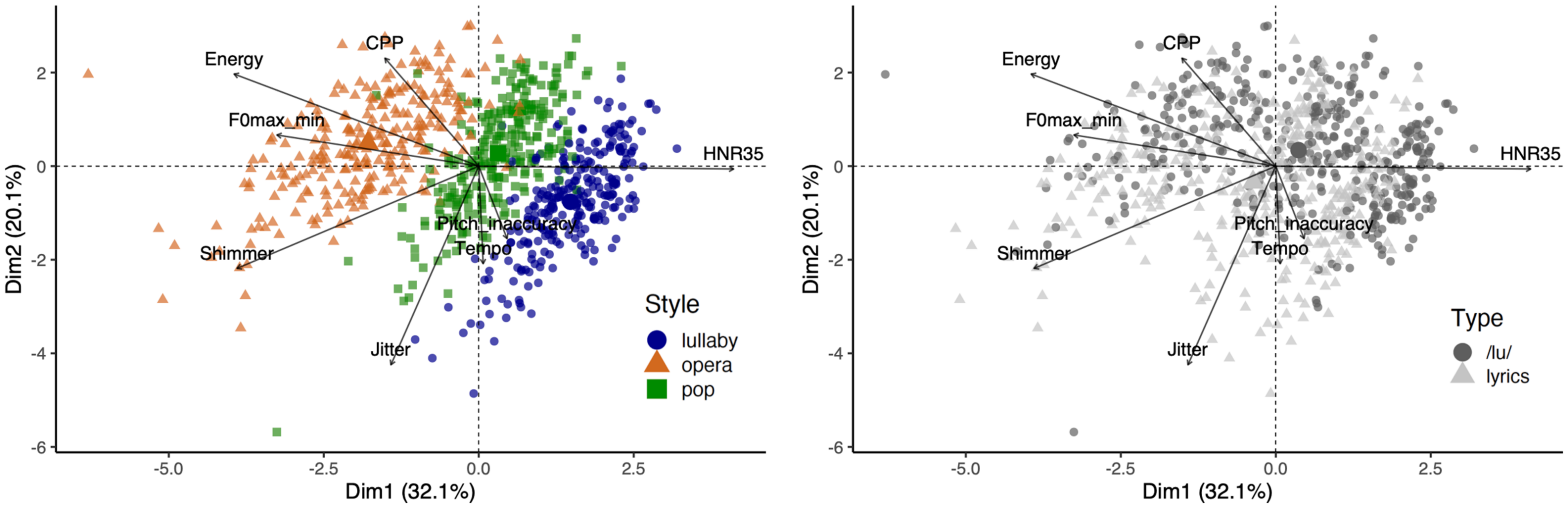
Biplots of principal component analysis showing singing performances as dots and loadings of acoustic features as arrowed vectors. Dots’ colors correspond to singing styles (left) or type of performance (right).

Results of the PCA based on the eight acoustic features are in line with the ANOVAs. Figure 3 presents visualizations of the singing performances along the first and second dimensions of the PCA (which explained 52.2% of the variance): very clear clusters are seen for performances in different styles of singing (Figure 3, left), but not for performances with different types of production (Figure 3, right). Please see the Supplementary Information for a scree plot (Supplementary Figure S3, top) and the contribution of each variable to the first, second and third dimensions of the PCA (Supplementary Figure S3, bottom). Note that we chose to include the Energy measure in this analysis because it is likely an important descriptor of the audio signal, but one can still recognize clear clusters of performances in different styles if one repeats the same analysis without the Energy measure (see Supplementary Figure S4).

The acoustic analysis presented here had the primary goal – and was able to – describe contrasting styles of singing performances. However, we acknowledge some important limitations: When recording stimuli, we aimed at obtaining naturalistic singing performances of high quality, and to focus on the acoustic signal itself. Our methodology did not follow practices customary in the field of voice science (e.g., Švec and Granqvist, 2010, 2018), where the primary interest is on voice production mechanisms. For instance, we adjusted microphone gain during recording to ensure good signal levels, while avoiding clipping. Further, we performed loudness normalization of stimuli of each style to different loudness levels, in line with the expected sound pressure level at production, that is, quiet for lullabies; intermediary for pop; and a lot louder for operatic performances. Whereas our choices aimed at ensuring recordings of good (artistic) quality, they also brought confounds to the interpretation of our acoustic measurements. More specifically, controlling sound pressure level is important to understand vocal function: increases in voice sound pressure level correlate with decreased jitter and shimmer, and increased HNR (Brockmann et al., 2008; Brockmann-Bauser et al., 2018) and CPP (Brockmann-Bauser et al., 2021). One further limitation is that operatic performances were recorded with higher pitch, which complicates comparisons with the other two styles because of influences of *𝑓ₒ* over other acoustic measures (e.g., Brockmann et al., 2011; Sampaio et al., 2020; Brockmann-Bauser et al., 2021; Baker et al., 2022). The use of vibrato (more pronounced in operatic singing) could also be associated with perturbation measures like jitter, shimmer and HNR (Larrouy-Maestri et al., 2014). Further, comparing naturalistic performances, which vary simultaneously in many dimensions, is obviously challenging. Approaches like ours should be complemented with other research designs, like case studies (e.g., Sundberg et al., 1993; Thalen and Sundberg, 2001; Stone et al., 2003) and studies using synthesized stimuli (e.g., Sundberg, 2006; Baker et al., 2022) where variables are changed (as much as possible) in isolation. One additional limitation is that, due to the large number of recorded takes, analysis was conducted on only about one third of all recorded singing material. Selection for analysis followed clear criteria (exclusion of performances with ambient noise, low vocal quality, low expressiveness and/or low authenticity), but despite efforts to maintain objectivity, some subjectivity is inherent in this selection process.

Despite these shortcomings, we indicate possible (functional) meanings of our measures and, more importantly, we report large differences between styles, suggesting singers’ proficiency in producing contrasting singing performances. On the other hand, different acoustic profiles do not necessarily mean that the performances will be perceived as intended. To clarify this point, we further investigated singers’ versatility in Part II, by looking at the perceptual experience of listeners when listening to these performances.

## 3. Part II: perception of singing performances

We conducted a behavioral experiment to examine whether the singing performances sounded as intended to naïve listeners. Recognition accuracy is thus used as a proxy to singers’ versatility – the rationale behind this is that versatile singers should be able to produce contrasting and characteristic-enough performances for participants to accurately recognize. By recruiting lay listeners, that is, participants without specific musical training, we aimed to examine participants with a large range of expertise, which is meant to be representative of a general population. Note that studies indicate that lay listeners are able to judge perceptual features of voices if adequate scales are used (Bänziger et al., 2014; Merrill, 2022). We assessed participants on a forced-choice task in which they had to indicate whether a given performance sounded like a lullaby, a pop song, or an opera aria.

### 3.1. Method

#### 3.1.1. Participants

Fifty participants (30 self-reported as female, 20 as male, *M* = 46.6 years old, *SD* = 17.2, 45 with German as mother tongue, from which 5 bilinguals, none of them with Portuguese as mother tongue) were recruited from the participant database of the Max Planck Institute for Empirical Aesthetics, in Frankfurt, Germany. They did not have hearing impairment and were mostly lay listeners. Participants were randomly assigned to one of two groups, which differed only in terms of which stimuli they were presented with (i.e., performances with lyrics for Group 1 and performances with a /lu/ sound for Group 2; see details in the Procedure section). According to an 18-items adapted version^8^ of the scale of music sophistication of Gold-MSI (Müllensiefen et al., 2014), the average music sophistication score was 81.4 (*SD* = 19.6) for Group 1 and 75.7 (*SD* = 13.7) for Group 2 (these values are not statistically different, two-samples *t*(42.9) = 1.2, *p* = 0.239). Participants were compensated at the rate of 14€ per hour of participation.

#### 3.1.2. Material

The stimulus set consisted of the 788 performances, that is, six melody excerpts performed by 22 singers, in three styles, with lyrics or a /lu/ sound, as described in Part I.

#### 3.1.3. Procedure

The experiment was implemented in Labvanced (Finger et al., 2017). The experimental procedure was ethically approved by the Ethics Council of the Max Planck Society, and was undertaken with written informed consent of each participant.

The session began with oral and written instructions, followed by four practice trials with example stimuli which were not part of the final stimulus set, presented through headphones (Beyerdynamic DT 770 PRO 80 Ohm), at a volume adjusted to a comfortable level. In each trial, participants were instructed to indicate if the stimulus presented sounded like a lullaby, a pop song, or an opera aria, by clicking on the respective answer. One group of participants (Group 1, *N* = 25) was presented only with performances with lyrics (395 trials) and one group of participants (Group 2, *N* = 25) only with performances with /lu/ (393 trials). For each group, stimuli from different melodies and styles were presented intermixed and in random order. The visual display of response alternatives (“as a lullaby,” “as a pop song,” “as an opera aria”) was presented in all possible orders but the order was fixed for each participant across the whole session. The experiment was divided into six blocks of 66 trials [except for the last block, which was slightly shorter due to a few missing stimuli (see Footnote 5)], and participants could take a break between blocks. The testing session lasted between 85 and 120 min. Each stimulus was presented once, except for 20 repetitions of a random subset of stimuli in the end of the experiment (different for each participant), which were used to estimate the test–retest intrarater agreement. At the end of the session, participants completed the adapted version of the general music sophistication scale of the Goldsmiths Music Sophistication Index (Müllensiefen et al., 2014).

#### 3.1.4. Statistical analyses

##### 3.1.4.1. Accuracy of style recognition

To test if singing styles were recognized, we compared the proportion of correct responses (across all participants) in each style against chance level (33%), with *Z*-tests for proportions (one-tailed; with the R function prop.test; separately for performances with lyrics and with /lu/). The reported *p*-values have been adjusted with the R function p.adjust to control the family-wise error rate (FWER) of these 6 comparisons with the Holm method (Holm, 1979). To test if accuracy was similar for performances with lyrics (Group 1) and /lu/ (Group 2), we used a two-tailed *Z*-test for proportions. Additionally, for each group, we also compared styles pairwise with *Z*-tests for proportions (two-tailed; also here, reported *p*-values have been adjusted with the Holm method).

##### 3.1.4.2. Accuracy by singer and versatility in pop and lullaby singing

We calculated the proportion of accurate responses for each singer, both across all styles and by style. Since these were productions of classical singers, the proportion of accurate recognition of operatic performances was expected to be high. The proportion of correct recognition of pop and lullaby performances, on the other hand, was taken as indicative of singers’ versatility: the more versatile the singer, the more competent she would be in producing non-operatic performances.

##### 3.1.4.3. Versatility and musical training

We also explored the relationship between singers’ versatility (as measured by the proportion of correct recognition for each singer in the pop and lullaby styles) and singers’ characteristics such as age, years of voice training, years of instrument training, general music training (years formally studying music, that is, enrolled in an institution such as conservatory/university), proportion of time singing as a soloist versus in a choir, and average number of hours spent performing per week (including practicing). To do so, we fit one multiple linear regression model (with the lm function) for each style, predicting the proportion of correct recognition from singers’ characteristics.

##### 3.1.4.4. Intrarater agreement analysis

To assess the consistency of participants’ responses, we calculated the test–retest intrarater agreement. Based on a subset of 20 repeated trials at the end of the experiment, we calculated Cohens’ Kappa, using the kappa2 function from the irr package in R (Gamer et al., 2019). According to Landis and Koch (1977), Kappa values between 0 and 0.2 indicate slight agreement; between 0.21 and 0.40, fair agreement; between 0.41 and 0.60, moderate agreement; between 0.61 and 0.80, substantial agreement; and between 0.81 and 1, perfect agreement. We also report the simple percentage agreement (agree function from the same package). These values were computed separately for Groups 1 (performances with lyrics) and 2 (performances with /lu/). Note that due to a mistake in the coding of the experiment, for a subset of 10 participants of Group 2, the planned repeated trials were not in fact repeated trials, but stimuli with lyrics instead of /lu/. Because of this, computation of Kappa for Group 2 is based only on the 15 participants that were correctly presented with repeated trials.

### 3.2. Results and discussion

#### 3.2.1. High accuracy of style recognition

**Figure 4:**
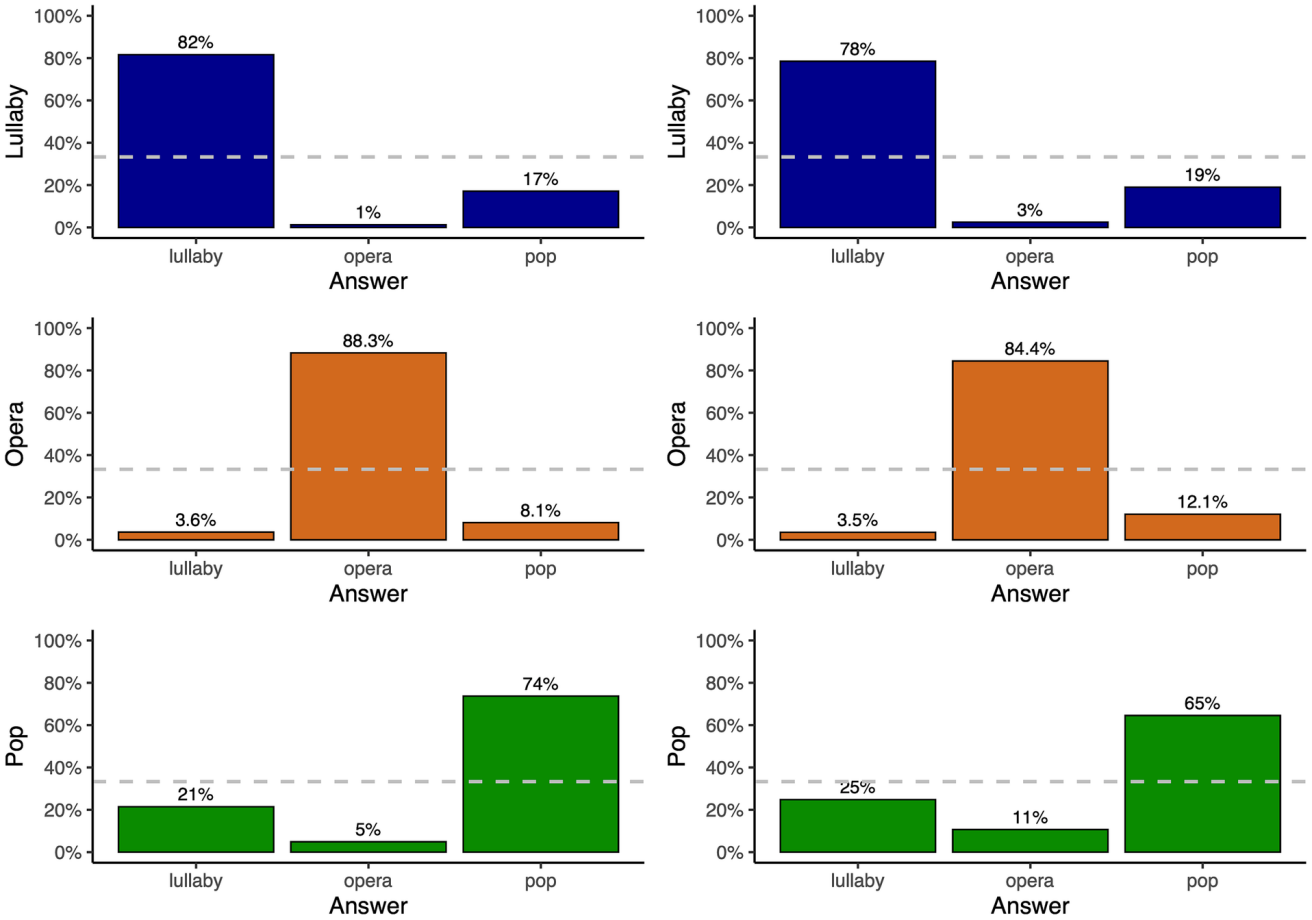
Classification of styles by participants in the perceptual experiment for performances with lyrics (left) and /lu/ (right), in trials where presented stimuli were lullaby (top), operatic (middle) or pop performances (bottom). In both panels, the dashed gray horizontal line represents chance-level performance.

The overall proportion of accurate responses was higher for performances with lyrics than for performances with /lu/ [81.2 and 75.8% respectively, *χ^2^*(1) = 82.9, *p* < 0.001], indicating that even though participants did not understand the lyrics of the melodies (performed in Brazilian Portuguese), they could still benefit from the phonetic content of performances when recognizing the style of singing. Note that, at the end of the experiment, participants of Group 1 (performances with lyrics) were asked if they recognized the language of the performances and about one fourth did. Five of them identified Portuguese, two Brazilian Portuguese, and one mentioned Eastern European language. Figure 4 illustrates the accuracy of recognition by style. Participants could recognize singing styles above chance level for all three styles: the proportion of accurate responses from Group 1 (performances with lyrics) was 88.3% for operatic performances [different from chance level, *χ^2^*(1) = 4456.2, *p* < 0.001], 81.6% for lullabies [*χ^2^*(1) = 3466.9, *p* < 0.001], and 73.7% for pop performances [*χ^2^*(1) = 2424.6, *p* < 0.001]. These values were different from each other: the proportion of accurate responses for operatic performances was higher than for lullaby [*χ^2^*(1) = 57.1, *p* < 0.001] and pop performances [*χ^2^*(1) = 226.6, *p* < 0.001], and it was higher for lullaby than for pop performances [*χ^2^*(1) = 59.5, *p* < 0.001]. The same pattern was found for Group 2 (performances with /lu/): the proportion of accurate responses was 84.4% for operatic performances [different from chance-level performance, *χ^2^*(1) = 3854.3, *p* < 0.001], 78.5% for lullabies [*χ^2^*(1) = 3033.4, *p* < 0.001], and 64.5% for pop [*χ^2^*(1) = 1426.5, *p* < 0.001] performances. Again, these values were different from each other: the proportion of accurate responses for operatic performances was higher than for lullaby [*χ^2^*(1) = 38.4, *p* < 0.001] and pop [*χ^2^*(1) = 340.3, *p* < 0.001] performances, and it was higher for lullaby than for pop performances [*χ^2^*(1) = 156.8, *p* < 0.001].

#### 3.2.2. Accuracy by singer and versatility

**Figure 5:**
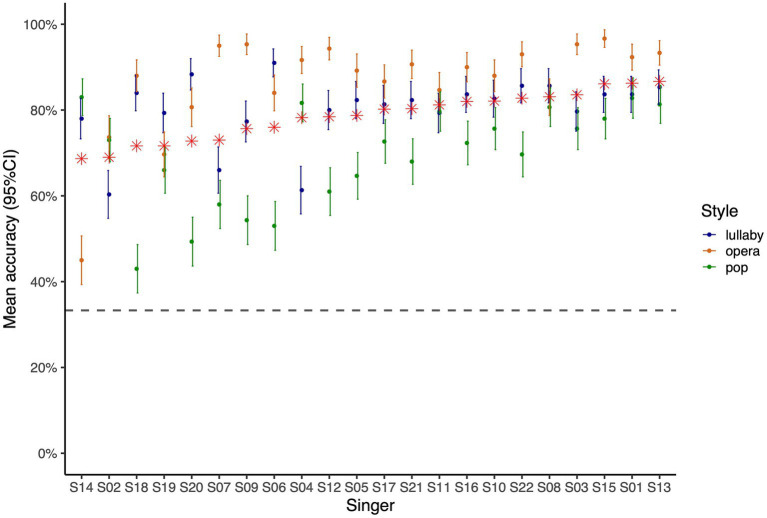
Proportion of correct style recognition across all 50 participants in the perceptual experiment for performances by each individual singer, as a proxy of singers’ versatility. Colors indicate singing styles. Red stars indicate the proportion of accurate recognition across the three singing styles. Error bars indicate 95% confidence intervals, and the horizontal gray dashed line corresponds to chance-level performance.

Figure 5 displays the proportion of correct recognition of performances produced by each singer. The overall proportion of correct recognition for performances by each singer was between 69 and 87%. In the case of operatic performances, accuracy ranged from 45 to 97% (or from 73.7 to 97% excluding singers S14 and S19, with declared vocal problems). Importantly, the productions of all singers in the lullaby and pop styles were recognized well above chance-level, showing singers’ versatility outside their strict field of classical training. For lullaby performances, the proportion of correct recognition was between 60 and 91%, and for pop performances, between 43 and 83%. Note the most common mistake made by participants was to answer that pop performances were lullabies (or *vice-versa*, to a slightly smaller extent).

#### 3.2.3. Versatility and musical training

Using the proportion of correct recognition of pop and lullaby performances as a proxy of singers’ versatility, we explored its relationship with singers’ characteristics such as years of voice and instrument training, formal music training (years enrolled in a conservatory or a music university), proportion of solo (to choir) singing, and average number of hours spent performing per week, *via* statistical modeling. Surprisingly, none of the mentioned variables predicted the proportion of correct recognition. In other words, classical singers’ ability to adjust or adapt (highly trained) motor schemas to perform in other styles was not affected by the extent of their musical training. Please see the Supplementary Information for a correlation matrix with all these predictors and the proportion of correct recognition by style (Supplementary Figure S5), as well as the coefficients of (non-significant) linear regression models predicting the proportion of correct recognition for each style from singer characteristics (Supplementary Figure S6). More detailed information about singers’ professional and private lives would be helpful for qualitative exploration of the impact of singers’ experiences on their versatility.

Interestingly, the ability to sound operatic (i.e., estimated by the proportion of correct recognition of operatic performances) was correlated with singers’ age (*r*_20_ = 0.44, *t* = 2.2, *p* < 0.05), suggesting that this typicality in operatic sounding might come with gathered experience rather than formal training itself. However, the lack of relationship between the proportion of recognition of operatic performances and singers’ (classical) music training might reflect a ceiling effect, since we purposely recruited highly trained singers, who presumably already had sufficient training to perform in this specific style.

#### 3.2.4. Intrarater agreement

Analysis of repeated trials showed participants were consistent in their responses: For Group 1 (performances with lyrics), analysis of test–retest intrarater agreement showed a simple percentage agreement of 82% (i.e., participants gave the same response at both the first and the second presentation of a given stimulus in 82% of the 20 trials), and a Kappa value of 0.73 (*z* = 22.9, *p* <. 001), indicating substantial agreement. For Group 2 (performances with /lu/), the simple percentage of agreement was 73%, and Kappa was 0.59 (*z* = 14.5, *p* < 0.001), indicating moderate agreement. Additionally, Supplementary Figure S7 (left) shows the proportion of correct recognition by each participant of this experiment. It ranged from 49.1 to 93.2%, indicating that, while there were individual differences in how well participants could do the task, all of them could do it above chance level, and the majority did it with good accuracy.

### 3.3. Control experiment

Our approach of normalizing stimuli of different styles to different loudness levels raised the question of how much participants’ high style recognition could be linked to differences in loudness between styles. To better understand the role of stimulus loudness in participants’ perception and evaluation of our stimuli, we conducted a control experiment in which all stimuli were normalized to the same loudness level.

#### 3.3.1. Participants

Ten additional participants (6 self-reported as female, 3 as male, 1 undisclosed, *M* = 49.8 years old, *SD* = 19.2, 9 with German as mother tongue, from which 3 bilinguals, none of them with Portuguese as mother tongue) were recruited from the participant database of the Max Planck Institute for Empirical Aesthetics, in Frankfurt, Germany. After completing the experiment, four participants answered they recognized the language used, but only one correctly responded Portuguese (one wrote “a fantasy language with elements of Portuguese,” one Spanish, one Italian). They did not have hearing impairment and were lay listeners, with an average music sophistication score of 88.5 (*SD* = 10.6) according to the same 18-items adapted version of the scale of music sophistication of Gold-MSI (Müllensiefen et al., 2014). Participants were compensated at the rate of 14€ per hour of participation.

#### 3.3.2. Material

We used half of the stimulus material of the main experiment, that is, a subset of 396 performances corresponding to three melodies (Nana Nenê, Chove Chuva, and Melodia Sentimental). Using the software To Audio Converter (version 1.0.16–1,059), all stimuli were loudness normalized (following the EBU-R128 standard) to −23 LUFS.

#### 3.3.3. Procedure

The only difference in procedure in relation to the original experiment was that all participants dealt with performances both with lyrics and with /lu/, though in different blocks of trials (in counterbalanced order). As before, stimuli from different styles were presented intermixed and in random order, and participants had to indicate if singing performances sounded like a lullaby, a pop song, or an opera aria in a forced-choice design. We also included 20 repeated trials at the end of respective blocks (10 trials for stimuli with lyrics and 10 for stimuli with /lu/) in order to conduct a test–retest intrarater agreement analysis.

#### 3.3.4. Statistical analyses

We repeated the analyses described for the first experiment: we compared the proportion of accurate responses for each style (across all participants) against chance-level performance (33% correct recognition) with *Z*-tests for proportions (one-tailed; separately for performances with lyrics and with /lu/; adjusting *p*-values to control the FWER with the Holm method). We also compared recognition between styles with pairwise *Z*-tests for proportions (two-tailed; separately for performances with lyrics and with /lu/; and adjusting *p*-values to control the FWER of 6 comparisons with the Holm method). Once again, to test if accuracy was similar for performances with lyrics and /lu/, we used a two-tailed *Z*-test for proportions. Finally, to compare results across experiments, we performed *Z*-tests for proportions for each style (two-tailed; separately for performances with /lu/ and lyrics; adjusting *p*-values to control the FWER of 6 comparisons with the Holm method). Additionally, we computed a Pearson correlation score between the overall proportion of correct recognition by stimulus item in both experiments. We also conducted analysis of test–retest intrarater agreement based on repeated stimuli.

### 3.4. Results and discussion

Analysis of repeated trials showed that participants of the control experiment were also consistent in their responses, with a simple percentage agreement of 81.5% and a Kappa value of 0.72 (*z* = 14.5, *p* <. 001), indicating substantial intrarater agreement. The proportion of correct recognition by each participant ranged from 43.9 to 88.4% (see Supplementary Figure S7, right), confirming that, as observed in the main experiment, recognition was above chance level for all participants (and the majority did the task with good accuracy).

Both for performances with /lu/ and with lyrics, recognition was above chance level for all styles (all *p*s < 0.001). For performances with /lu/, we found the same pattern as in the main experiment: higher recognition for operatic (81% CR) than lullaby singing [73% CR; *χ^2^*(1) = 13.92, *p* < 0.001] and pop singing [61% CR; *χ^2^*(1) = 64.59, *p* < 0.001] and higher for lullaby than pop singing [*χ^2^*(1) = 19.29, *p* < 0.001]. For performances with lyrics, the recognition of operatic performances (82% CR) was higher than that of lullabies [68.6% CR; *χ^2^*(1) = 30.77, *p* < 0.001] and pop [67.6% CR; *χ^2^*(1) = 35.42, *p* < 0.001], but there was no difference between recognition rates for pop and lullaby performances [*χ^2^*(1) = 0.17, *p* = 0.679]. Also, in this experiment there was no difference between overall recognition rates for performances with lyrics (72.7% CR) and /lu/ [71.8% CR; *χ^2^*(1) = 0.36, *p* = 0.546]. Please see Supplementary Figure S8 for the proportion of correct recognition by style, and Supplementary Figure S9 for a display of the proportion of correct recognition of performances by each singer in the control experiment.

When comparing recognition rates between experiments, we found that, for performances with lyrics, recognition was higher for all styles in the main experiment [lullaby: *χ^2^*(1) = 56.65, *p* < 0.001; opera: *χ^2^*(1) = 20.5, *p* < 0.001; pop: *χ^2^*(1) = 10.4, *p* < 0.01]. For performances with /lu/, recognition rates seemed higher in the main experiment for all styles, but this difference only reached significance for lullabies [*χ^2^*(1) = 10.46, *p* < 0.01; opera: *χ^2^*(1) = 3.82, *p* = 0.1; pop: *χ^2^*(1) = 2.38, *p* = 0.12]. The illustration of the overall proportion of correct recognition by stimulus item in both experiments can be found in Supplementary Figure S10. Values were highly correlated between experiments [*r*_(396)_ = 0.79, *p* < 0.001], suggesting consistency in how recognizable a given item was across experiments, that is, items that were well recognized in the main experiment were likely to be well recognized in the control experiment.

Overall, the slightly higher proportion of correct recognition in the main experiment suggests that the difference in loudness levels between styles might have aided style recognition in that experiment. However, the high proportion of recognition in the control experiment suggests that the difference in loudness levels was not essential for correct style recognition. In other words, singing performances in different styles were contrasting enough, so that other perceptual features could inform participants’ style recognition. Readers interested in the role of acoustic features in the perceptual categorization of different singing styles are invited to read the Supporting Text 2 in the Supplementary materials, where we describe an additional exploratory analysis on this subject (illustrated in Supplementary Figures S11, S12).

## 4. General discussion

The contrasting acoustic profiles of melodies performed as a lullaby, as a pop song, or as an opera aria, aligned with the high recognition of their intended styles by lay listeners, indicate that classical singers were highly versatile. They not only performed as expected in the style in which they were trained, but managed to refrain from using this specific technique (or arguably, to adapt it) to sing in contrasting styles.

The acoustic analysis showed different acoustic profiles for the three described singing styles, but, as mentioned before, has limitations that may hinder insights about differential mechanisms of production. The acoustic profile of operatic singing included slower tempo, extensive use of vibrato, higher shimmer, lower harmonics-to-noise ratio, and intermediary CPP values. Lullabies had reduced use of vibrato, higher jitter (possibly related to their soft phonation level – e.g., Brockmann et al., 2008, 2011) and lower CPP (likely related to a breathy voice quality – e.g., Murton et al., 2020), as well as worse pitch accuracy. Lullabies were also slower than pop performances. These measures combined suggest that singers did prioritize producing intimate, soft singing, over their usual classical voice production pattern. In the case of pop, performances were faster and had higher CPP values than both other styles.

The versatility of our cohort of classically trained singers was confirmed by the results of the perceptual experiment (replicated in the control experiment). Given their intensive training, it is not surprising that classical singers could provide “operatic sounding” recordings that were recognized as such (86.4% correct recognition). Their versatility is best expressed in the high recognition accuracy of lullaby (80%) and pop (69.1%) performances. Such high recognition rates might be enhanced by the choice of the task (forced-choice) and should be confirmed with a less constrained task (e.g., free label, see Fink et al., 2023). Note that the lower recognition accuracy for pop performances might reflect singers’ reduced experience in that style, but also uncertainty about what type of sound to produce, given the lack of a clear definition for pop singing. The majority of mistakes corresponded to participants answering that pop performances were lullabies (or *vice-versa* to a slightly smaller extent). In our study, these two styles were performed with the same pitch, so discriminating between them was indeed more difficult. Nevertheless, participants were able to correctly recognize these performances well above chance level. This was also the case in the control experiment, in which all performances were presented in the same loudness level. The fact that recognition rates were slightly lower in this experiment suggests that the difference in loudness levels probably assisted participants in recognizing styles in the first experiment. However, other perceptual aspects of the singing performances were salient and contrasted enough to inform participants’ decisions, allowing them to still recognize styles with high accuracy.

An interesting finding was that the proportion of correct recognition of operatic performances correlated positively with singers’ age (*r* = 0.44), but not with their musical training, suggesting that maturity and general experience as a classical singer influenced the recognition of their performances as operatic. According to Fitts and Posner’s theory of motor learning, after extensive practice, a performer will usually reach the autonomous phase, where movements are fluent, accurate and consistent, and movement production is efficient and requires little muscular energy (Fitts and Posner, 1967). At this stage, the skill is performed largely automatically and movement execution demands little to no attention (Fitts and Posner, 1967; Wulf, 2012). Considering the extensive motor learning involved in the years of training required to master the classical singing technique, it is remarkable that classical singers were able to adapt their performances to produce recognizable performances in other singing styles.

Importantly, some singers were more versatile than others when performing in styles outside their classical training, with large differences in the proportion of correct recognition of performances by different singers – ranging from 60 to 91% for lullaby and from 43 to 83% for pop performances (from 50 to 85% for lullaby and from 38 to 87% for pop performances in the control experiment). We investigated the relationship between singers’ demographics, musical training and practice and the proportion of correct recognition of singers’ performances in different styles. We found no relationship between these variables and singers’ versatility when singing in pop and lullaby styles. In other words, the amount of classical training did not seem to enhance singers’ versatility in pop and lullaby singing. This finding is in line with the point made by vocal pedagogues concerned with the limitations of the standard classical singing training in face of a highly dynamic, challenging and competitive job market (e.g., LeBorgne and Rosenberg, 2021).

One limitation of our study is that we only analyzed around one third of all recorded singing material. While it is conceivable that results might vary with analysis of the full singing material, we do not anticipate a significant impact – our (admittedly subjective) observation was that most singers were consistent in their productions, that is, that repeated takes within each condition did not vary much. This consistency is not surprising considering that singers were highly trained and performing at a professional level. A different concern relates to the authenticity of the recorded performances. In future investigations, it would be desirable to clarify how well classical singers can produce not only recognizable, but also stylistically authentic performances in different styles. A truly versatile singer should be able to produce performances that surpass mere resemblance to a certain stylistic reference. In other words, versatile singers should manage to produce not only stereotypical, but also genuinely convincing performances with artistic quality. An obvious first step could be to have expert judges evaluate our stimulus set in terms of quality and authenticity. This would involve the further challenges of identifying suitable expert judges and establishing corresponding criteria to evaluate the quality and authenticity of performances in each style. One other point worth exploring would be the role of singers’ life experiences in their versatility. All singers in this study declared having experience singing in other styles and were professional singers in Brazil, which requires flexibility (e.g., performing at weddings, teaching both classical and popular singing to attract more interested students). It would be interesting to further explore the role of singers’ professional experiences (e.g., teaching children), personal experiences (e.g., motherhood; broad music listening habits), and even personality traits (e.g., Costa and McCrae, 1992) in their versatility. Besides clarifying the benefits and limits of intense training, understanding the role of singers’ characteristics and diversity of training would also be important from a pedagogical point of view, in order to help singers who are not (yet) very versatile to improve this ability.

## Data availability statement

The original contributions presented in the study are publicly available. This data can be found here: https://osf.io/6eyuc/.

## Ethics statement

The experimental procedures of the studies involving humans were ethically approved by the Ethics Council of the Max Planck Society. The studies were conducted in accordance with the local legislation and institutional requirements. The participants provided their written informed consent to participate in this study.

## Author contributions

CB and PL-M conceived the study. CB recorded singers, collected data for the perceptual experiments, carried out the statistical analyses and data visualizations, and wrote the first draft of the manuscript. PL-M supervised all mentioned stages and revised the manuscript. All authors contributed to the article and approved the submitted version.

## Footnotes

^1^A note about singers’ vocal health: we did not explicitly ask for information about vocal health, but two singers declared having acute voice issues in the recording day: singer S14 reported having an acute allergy crisis. Singer S19 only mentioned her voice was “tired”; as the recording session progressed her voice became breathier and she had many voice cracks. Both singers insisted on completing the recording session, so we proceeded. We chose to include their recordings since they added variability. We repeated all analyses without the data from these two singers and found similar results – except that the proportion of correct recognition of operatic performances increased slightly from 86.4 to 89.3% (the difference for the other styles was of less than 0.5%).

^2^We considered that operatic singing typically has higher pitch than pop and lullaby singing. We address the implications of this choice in all comparisons across styles made throughout the study.

^3^A recording technician additionally adjusted the gain of the microphone for performances in different styles to maintain good signal rates and avoid clipping, since performances varied greatly in terms of sound intensity level.

^4^The complete stimulus set, including the speech vocalizations, will be made available via open access in a separate publication. We limit ourselves here to the singing performances, which will also serve as material of the perceptual experiment described in Part II.

^5^Unfortunately, nine performances were missing due to technical issues during the recording sessions (S01_NR_pop_U, S01_BO_pop_U, S05_AL_cl_L, S05_AL_cl_U, S07_NR_pop_L, S07_NR_pop_U, S09_NR_pop_L, S09_NR_pop_U; S11_NN_cl_U) but were replaced by additional recordings of good quality sent afterward by the cooperative singers, who recorded themselves a cappella in private settings. Note that 788 of them (395 with lyrics and 393 with /lu/) were used in Part II (i.e., in the perceptual experiment) since four additional recordings were received after data collection.

^6^http://www.phonetics.ucla.edu/voicesauce/documentation/parameters.html

^7^Note the RMS energy values reported here are a direct consequence of our loudness normalization of stimuli to different levels. At production, operatic singing was a lot louder than both others styles, and pop singing was louder than lullabies. Our manipulation kept the general loudness characteristics of stimuli to arbitrary loudness levels that sounded natural to the present authors, but since we varied distance to microphone and microphone gain during recording, we do not report any objective measure of intensity here.

^8^We included the following items: AE_01, AE_02, AE_05, AE_07, EM_04, MT_03, MT_07, PA_01, PA_04, PA_06, PA_07, PA_08, SA_01, SA_02, SA_03, SA_04, SA_05, SA_06. These correspond to 15 items from the general sophistication scale, plus three items corresponding to a short scale of perceptual abilities.
