# Supplementary material for: Classical singers are also proficient in non-classical singing
Source: Front Psychol. 2023 Oct 25;14:1215370. doi: 10.3389/fpsyg.2023.1215370 (PMC10630913; doi:10.3389/fpsyg.2023.1215370)
Supplement: Supplementary file 1 [file Data_Sheet_1.pdf]

Supplementary Materials

Classical singers are also proficient in non-classical singing

Bruder, Camila*; Larrouy-Maestri, Pauline

*** Correspondence:** camila.bruder@ae.mpg.de

Data availability

The raw data from both experiments and analyses code (.Rmd files and corresponding .html renderings), as well as examples of the singing performances, can be found at https://osf.io/6eyuc/.

Supplementary Table 1: Summary descriptive statistics of singers' characteristics.

	**Mean**	***SD***	**Min**	**Max**
**Singer age (years)**	32.50	7.10	22.00	51.00
**Years voice training**	12.93	6.00	4.50	27.00
**Years music training**	15.71	7.28	4.00	30.00
**Years instrument training**	4.25	3.89	0.00	15.00
**Hours performed per week**	15.96	9.81	1.00	40.00

	N	**Percentage**
**Proportion solo:choir singing**	(22)	
**1 (100% solo)**	5	22.7%
**2 (75% solo, 25% choir)**	5	22.7%
**3 (50% solo, 50% choir)**	5	22.7%
**4 (25% solo, 75% choir)**	7	31.8%
**5 (100% choir)**	0	-

Nana Nenê (anonymous)

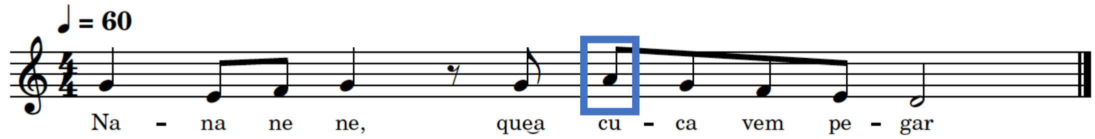

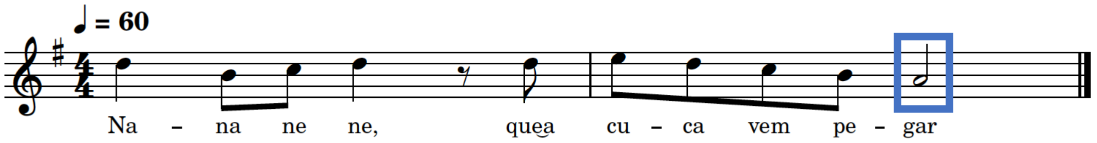

Boi da Cara Preta (anonymous)

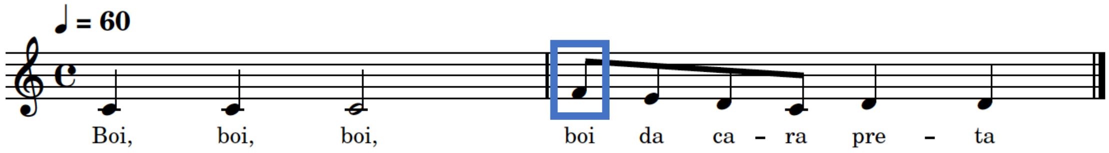

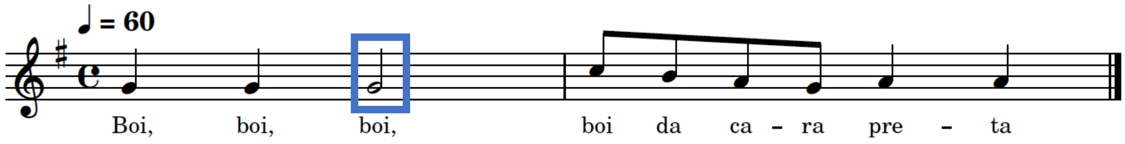

Alecrim Dourado (anonymous)

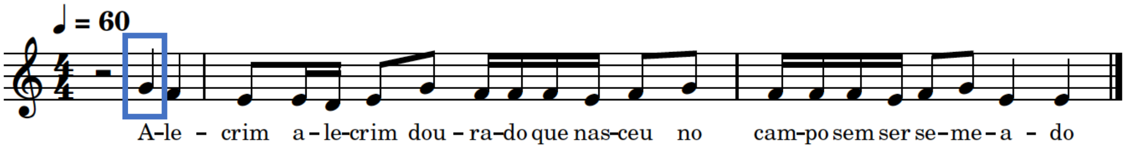

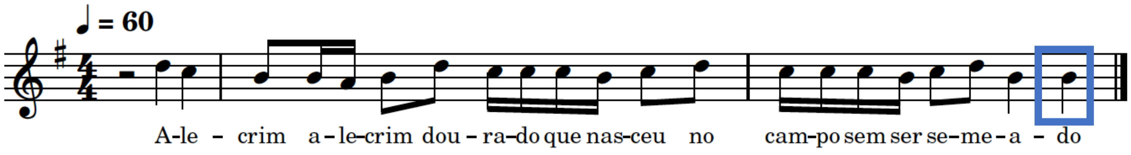

Nesta Rua (anonymous)

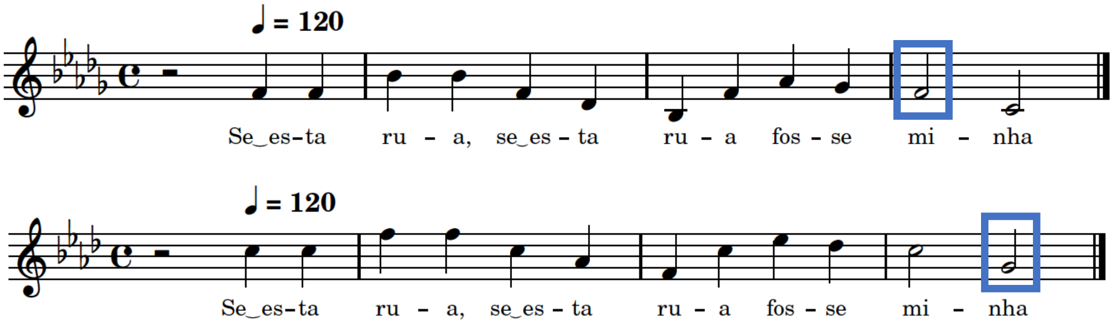

Chove Chuva (Jorge Ben Jor)

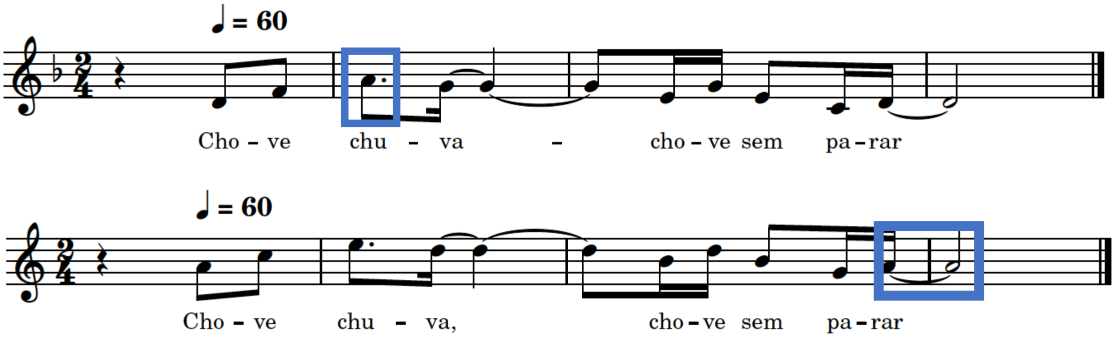

Melodia Sentimental (Heitor Villa-Lobos)

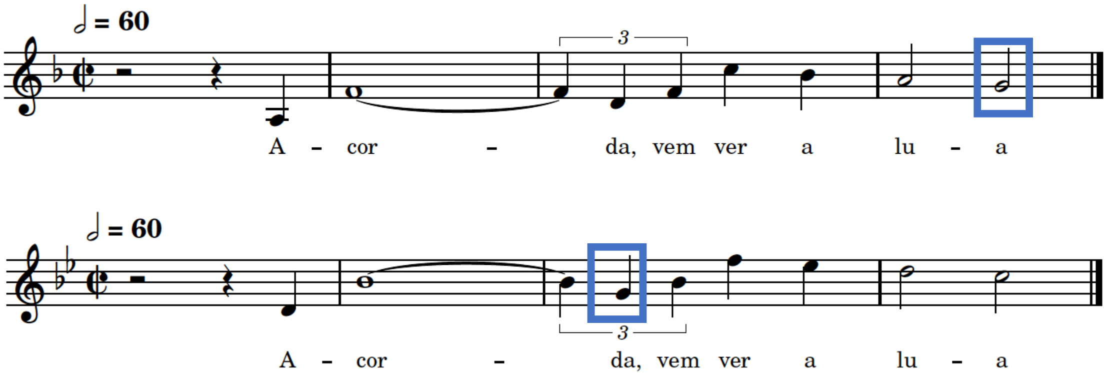

Supplementary Figure 1: Musical notation for the melody excerpts recorded. The first version of each melody was used for pop and lullaby versions, the second was transposed a fourth or fifth higher for operatic version. The notes marked with a blue frame were used for our proxy measure of vibrato extent, $f_o$max-min.

Supporting Text 1

Translation of the texts of performed melody excerpts

Nana nenê, que a cuca vem pegar
Sleep, baby, (or) the Cuca will come get.

Boi, boi, boi, boi da cara preta
Ox, ox, ox, black-faced ox

Alecrim, alecrim dourado que nasceu no campo sem ser semeado
Rosemary, golden rosemary that was born in the field without being sown

Se esta rua, se esta rua fosse minha
If this street, if this street were mine

Chove chuva, chove sem parar
It's raining rain, raining non-stop

Acorda, vem ver a lua
Wake up, come see the moon

Supplementary Table 2. Summary descriptive statistics for acoustic measures of stimuli by style. CPP: Cepstral peak prominence; RMS: Root Mean Square; HNR35: harmonics-to-noise ratio (0 - 3.5 kHz).

	Lullaby		Opera		Pop	
	**Mean**	**SD**	**Mean**	**SD**	**Mean**	**SD**
**CPP (dB)**	18.455	1.775	21.134	1.148	22.951	1.526
**RMS Energy (dB)**	0.874	0.125	5.94	1.536	3.198	0.689
**HNR35**	49.866	5.665	32.291	5.18	48.33	5.432
**Pitch inaccuracy (cents)**	29.742	18.437	25.975	14.449	26	16.257
**Tempo (bpm)**	69.956	12.314	65.834	10.33	74.747	13.818
**$f_o$max-min (Hz)**	22.22	10.608	47.826	26.523	26.836	15.456
**Jitter (local)**	0.004	0.001	0.003	0.001	0.003	0.001
**Shimmer (local)**	0.024	0.005	0.033	0.009	0.027	0.006

Supplementary Table 3. Summary descriptive statistics for acoustic measures of stimuli by type of production. CPP: Cepstral peak prominence; RMS: Root Mean Square; HNR35: harmonics-to-noise ratio (0 – 3.5 kHz).

	/lu/		Lyrics	
	**Mean**	**SD**	**Mean**	**SD**
**CPP (dB)**	20.408	2.5	21.285	2.175
**RMS Energy (dB)**	3.465	2.438	3.21	2.126
**HNR35**	46.698	9.621	40.294	8.509
**Pitch inaccuracy (cents)**	27.36	17.513	27.119	15.518
**Tempo (bpm)**	66.297	11.002	74.06	13.209
**$f_o$max_min (Hz)**	32.367	23.29	32.221	20.221
**Jitter (local)**	0.003	0.001	0.004	0.001
**Shimmer (local)**	0.026	0.007	0.03	0.008

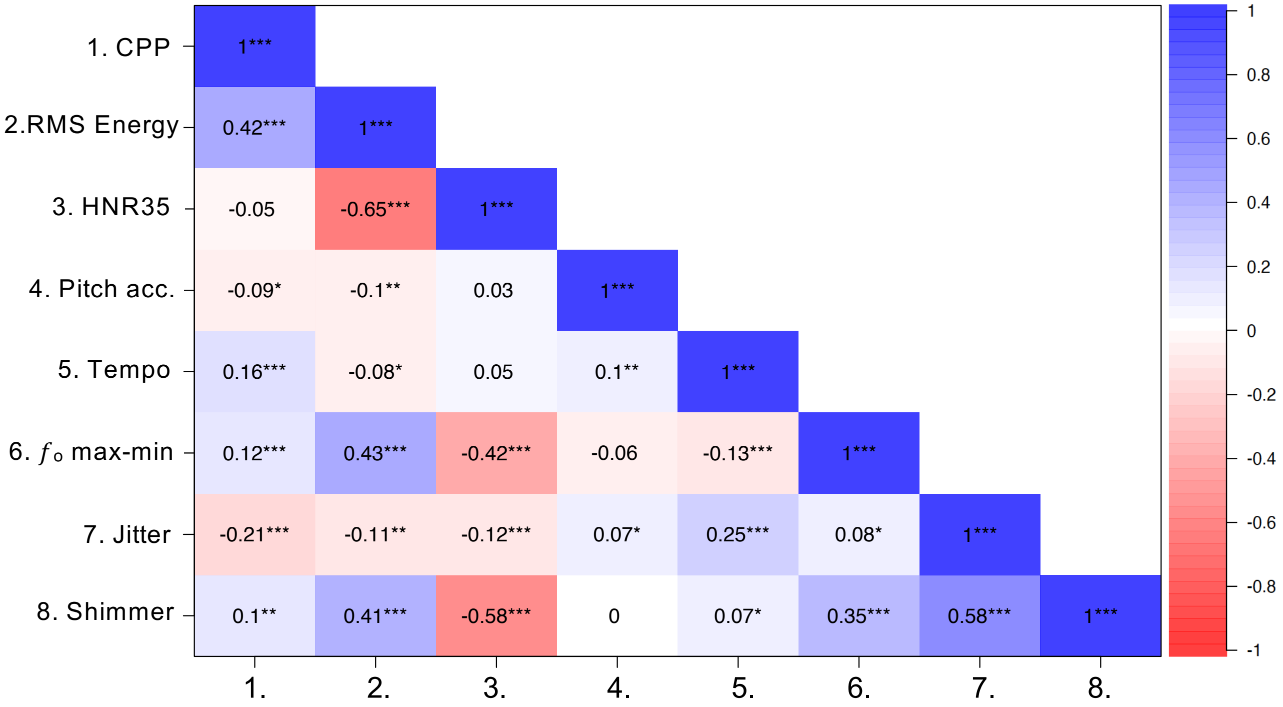

Supplementary Figure 2. Correlation matrix displaying Pearson correlation coefficients between the acoustic features measured. CPP: Cepstral peak prominence; RMS: Root Mean Square; HNR35: harmonics-to-noise ratio (0 – 3.5 kHz); Pitch acc.: pitch accuracy. Asterisks indicate significance of correlation without any correction for number of comparisons, since this analysis is exploratory/descriptive (*** $p < .001$; ** $p < .01$, * $p < .05$).

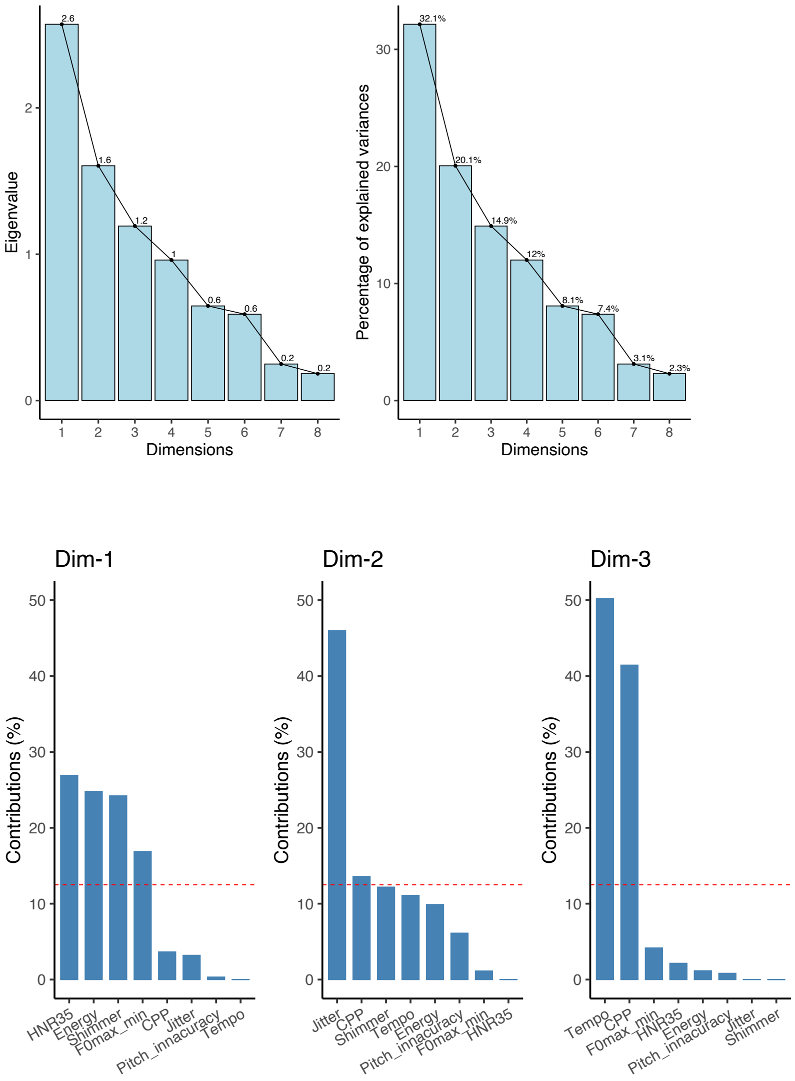

Supplementary Figure 3. Details of the Principal Components Analysis on the eight acoustic features. **Top:** Scree plot illustrating the eigenvalues for each component. The first two dimensions combined explain 52% of the variance; the first three dimensions combined explain 67% of the variance. **Bottom:** Contribution of each acoustic feature to the first, second and third dimensions of the PCA.

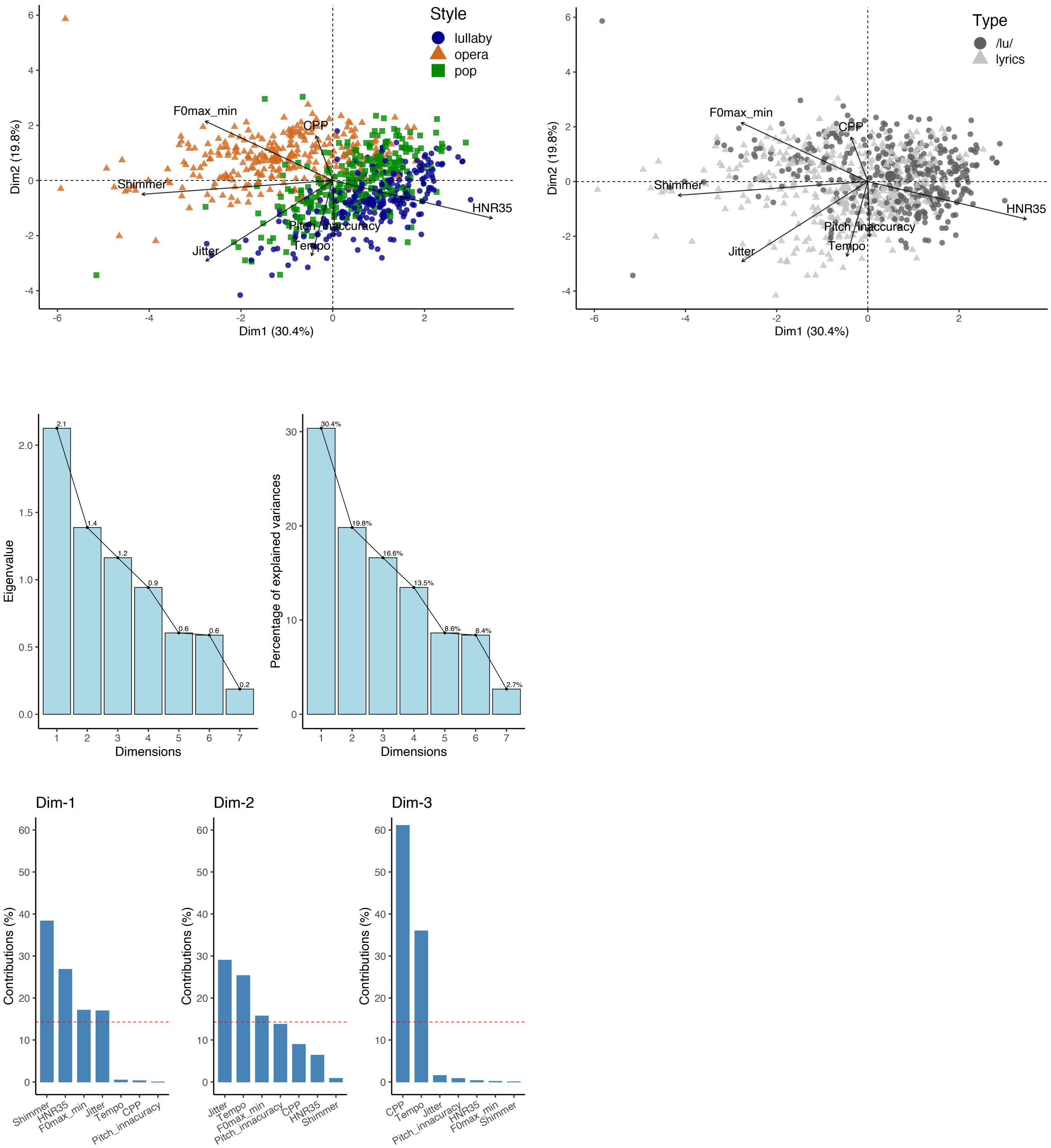

Supplementary Figure 4: Principal component analysis similar to the one shown in Supplementary Figure 3, but not including the Energy measure. **Top:** Biplots showing singing performances as dots and loadings of acoustic features as arrowed vectors. Dots' colors correspond to singing styles **(left)** or type of performance **(right)**. **Middle:** Scree plot illustrating the eigenvalues for each component. The first two dimensions combined explain 50.2% of the variance; the first three dimensions combined explain 66.8% of the variance. **Bottom:** Contribution of each acoustic feature to the first, second and third dimensions of the PCA.

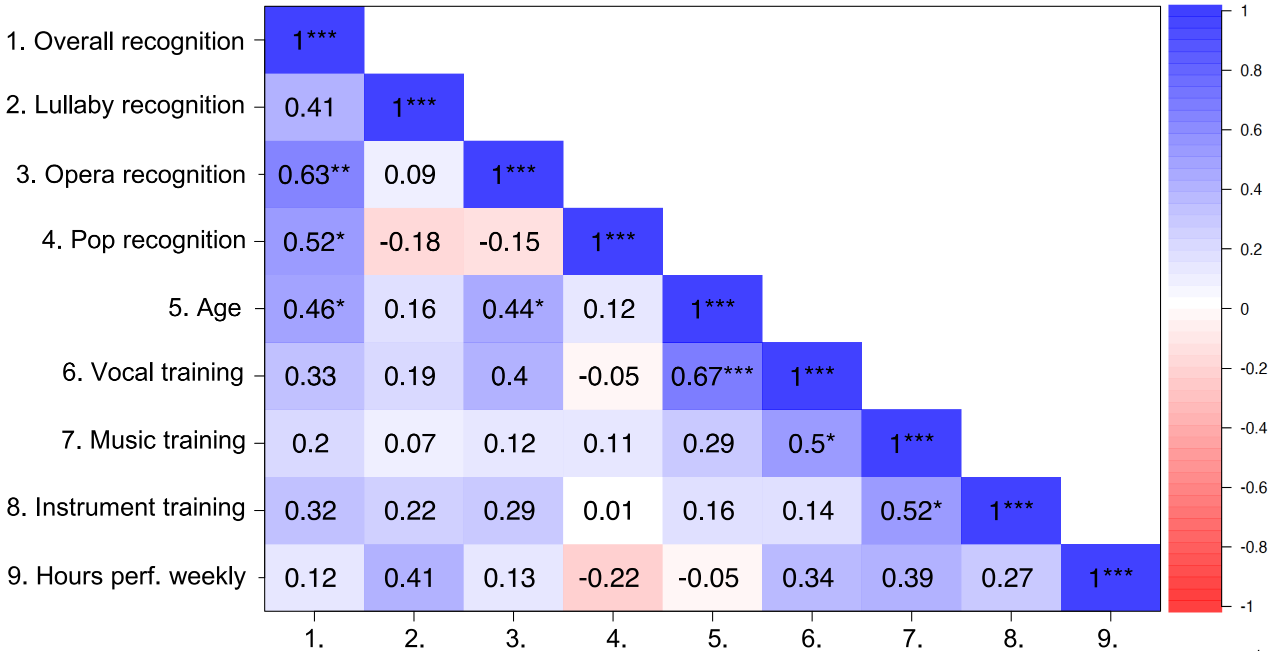

Supplementary Figure 5: Correlation matrix displaying Pearson correlation coefficients between the mean proportion of correct style recognition ("versatility") aggregated across all styles or computed separately for each style and singers' characteristics: age, years of voice training, years of formal musical training (in a conservatory or a music university), years of instrument training, average number of hours spent performing per week (including practice time). Note that all of these measures of musical training should increase as singers get older and accumulate training, causing them to correlate with each other. Asterisks indicate significance of correlation without any correction for number of comparisons, since this analysis is descriptive. *** $p < 0.001$; ** $p < 0.01$, * $p < 0.05$.

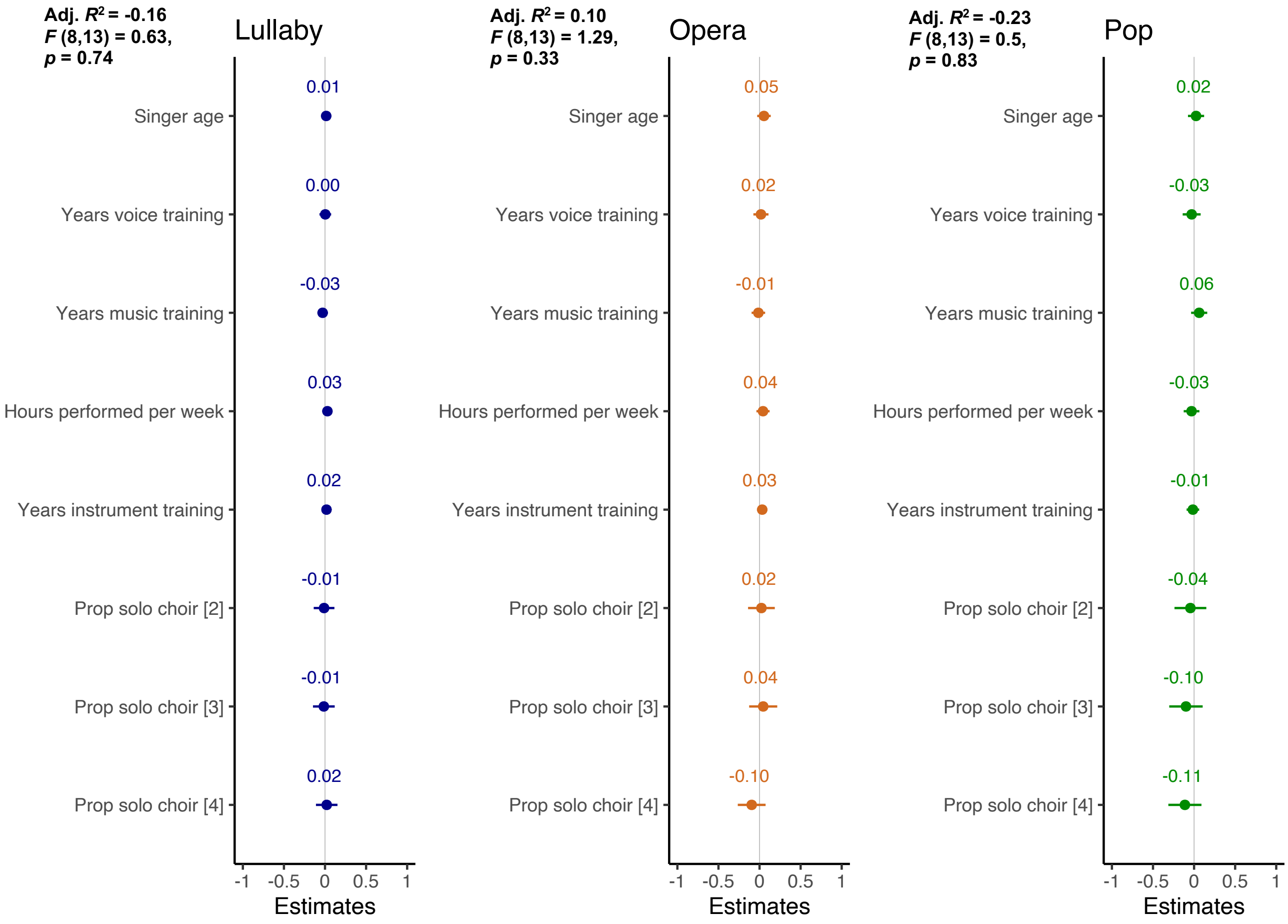

Supplementary Figure 6: Plots of coefficients of regression models predicting the proportion of accurate recognition for each style from singers' age, years of voice/music/instrument training, average number of hours spent performing per week and proportion of solo/choir singing. Note the categorical predictor proportion of solo/choir singing was coded in the same way as in Supplementary Table 1 (1 = 100% solo activity; 2 = 75% solo; 3 = 50% solo; 4 = 25% solo), with 1 (100% solo activity) as the group of reference in the present analysis. Coefficients are standardized by one standard deviation and displayed above their 95% confidence intervals. As reported for each style, none of the models reached significance and the variance explained was particularly low. Note that after step-wise reduction, the model for lullaby was not significant (F (1,20) = 4.1, p = .057; Adj. R^2 = 0.13) and only included as predictor the average hours of weekly performance (β = .03, p = .057). The model for opera was significant (F (1,20) = 4.8, p = .04; Adj. R^2 = 0.15) and only included singers' age as predictor (β = 0.05, p = .04). The model for pop did not include any predictor at all.

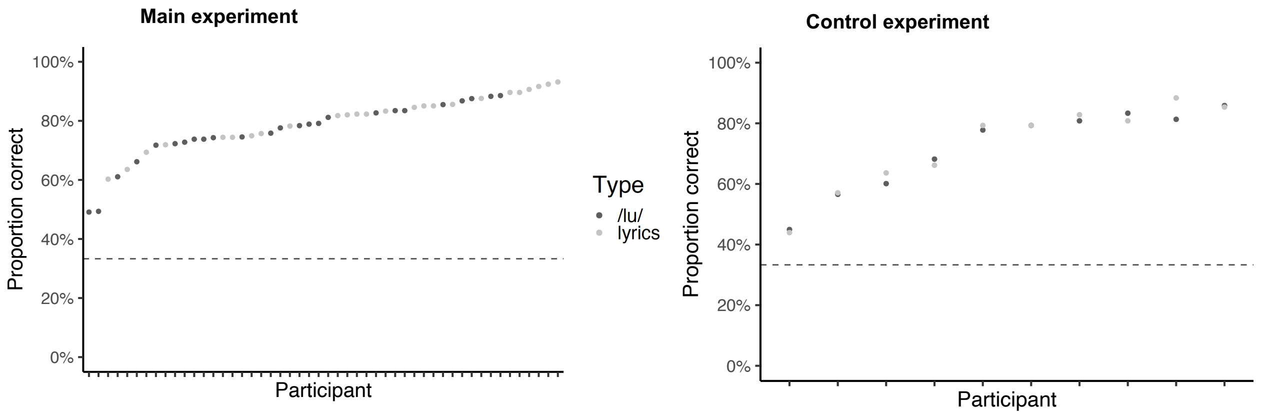

Supplementary Figure 7: Proportion of correct recognition by each participant of the main experiment (**left**; $N = 50$ participants, 788 stimuli; recognition rate of each participant represented by one dot) and of the control experiment (**right**; $N = 10$ participants, 396 stimuli; recognition rate of each participant represented by two dots). Colors indicate the type of stimulus (light gray for performances with lyrics, dark gray for performances with /lu/).

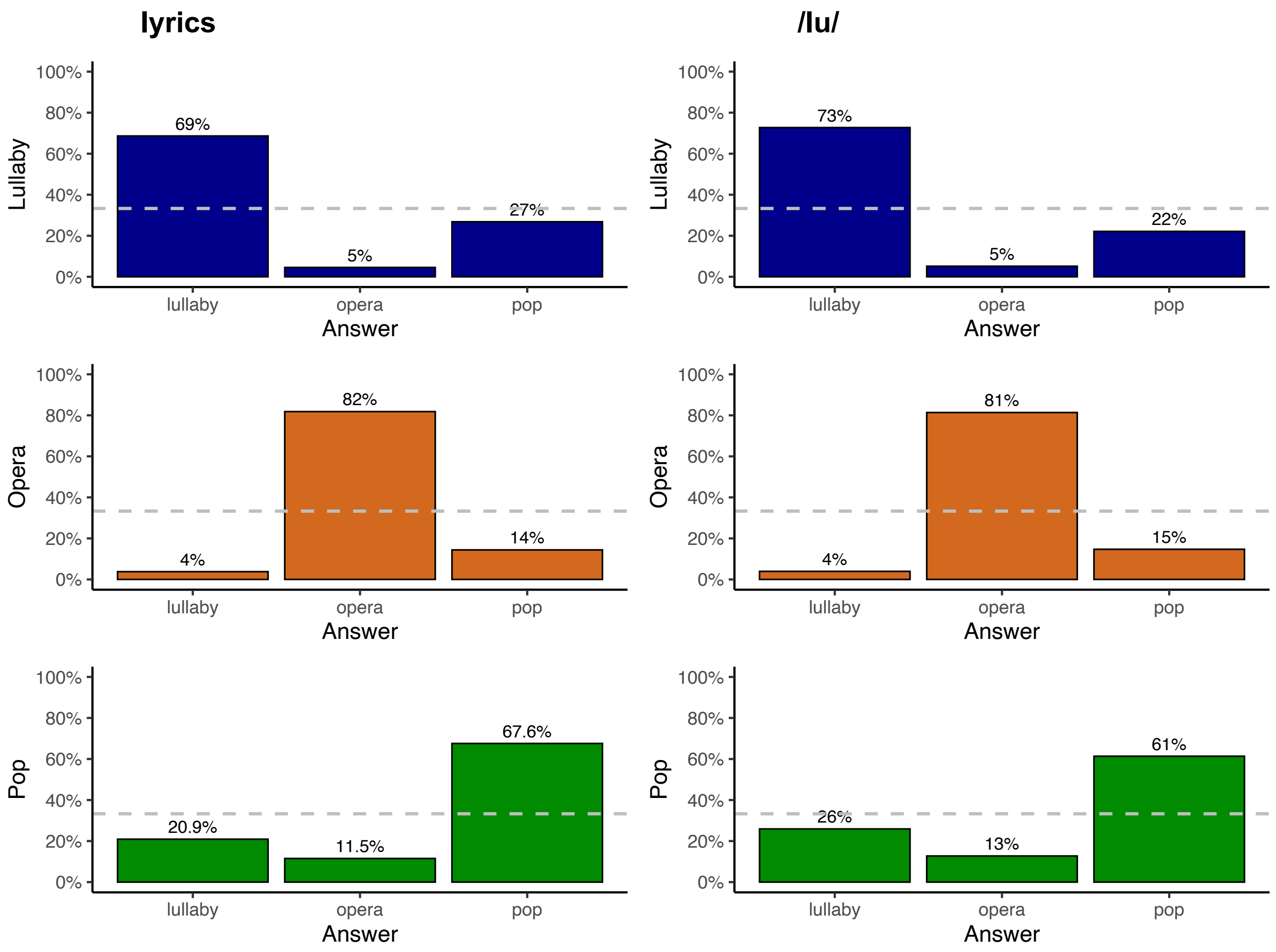

Supplementary Figure 8: Classification of styles by participants in the control experiment ($N = 10$) for performances with lyrics **(left)** and /lu/ **(right)**, in trials where presented stimuli were lullaby **(top)**, operatic **(middle)** or pop performances **(bottom)**. In both panels, the dashed gray horizontal line represents chance-level performance.

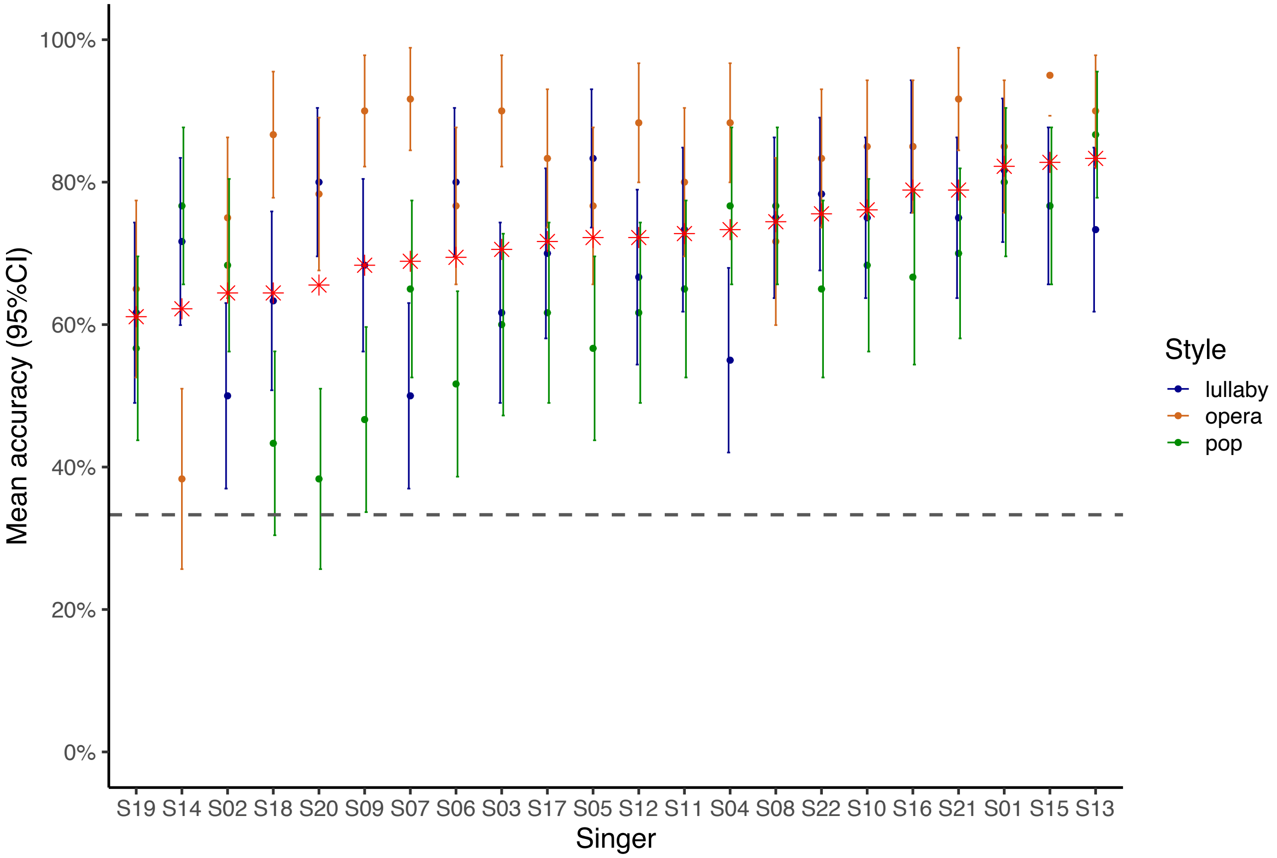

Supplementary Figure 9: Proportion of correct style recognition across 10 participants in the control experiment for performances by each individual singer. Colors indicate singing styles. Red stars indicate the proportion of accurate recognition across the three singing styles. Error bars indicate 95% confidence intervals, and the horizontal gray dashed line corresponds to chance-level performance.

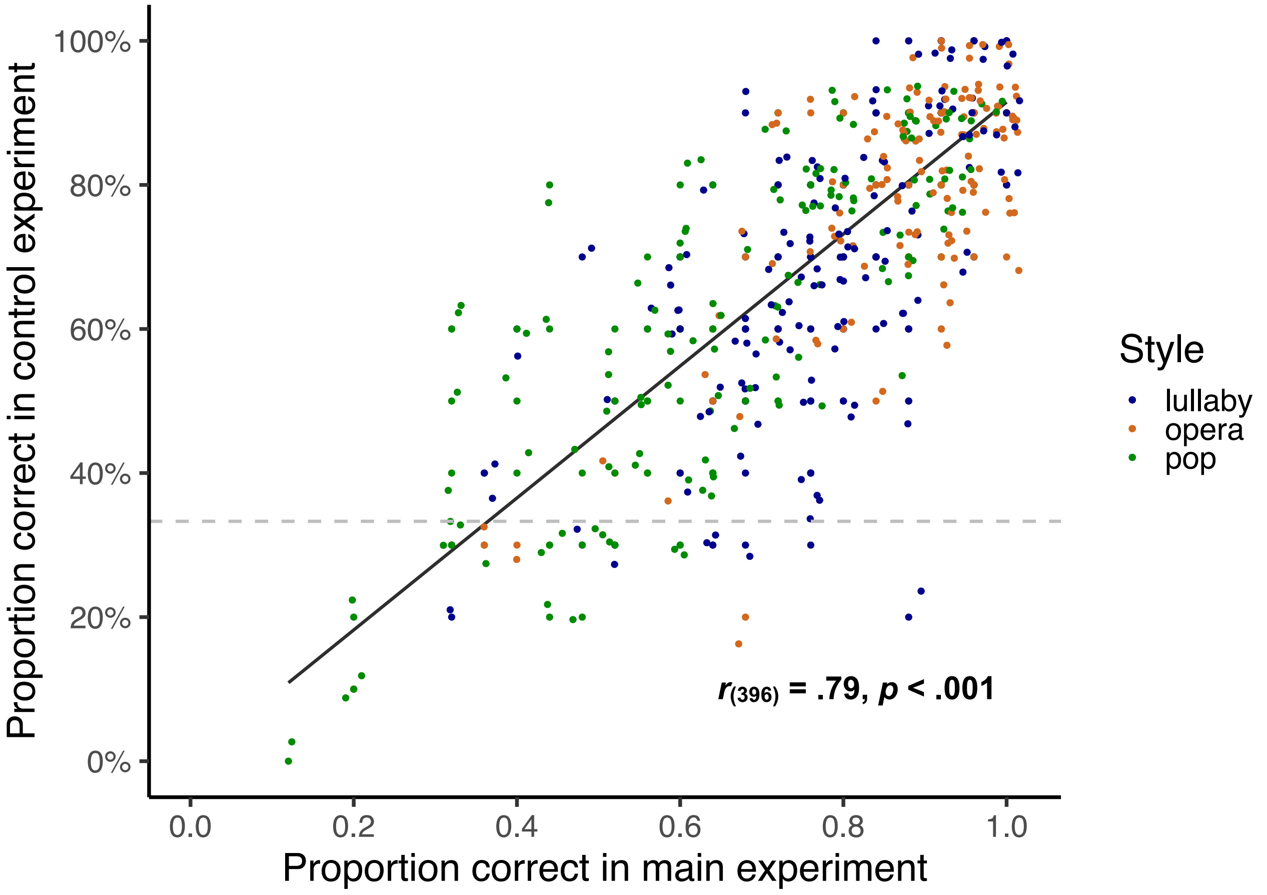

Supplementary Figure 10: Scatterplot of proportion of correct style recognition of each stimulus item in the main experiment ($N = 50$ participants) and in the control experiment ($N = 10$). Each dot represents one stimulus ($N = 396$ stimuli), and colors indicate singing styles.

Supporting Text 2

Exploratory analysis: role of acoustic features on perceptual categorization of different singing styles

We built separate linear regression models for each style, where the dependent variable was the overall proportion of correct stimulus recognition (computed for each stimulus across all participants) and the predictors were the acoustic features. All models were significant (*p*s < .001) and could account for some of the variance of the dependent variable (adjusted R^2 =.17 for lullaby, .34 for pop and .39 for opera). We would argue that these models indicate which acoustic features were most relevant for correct recognition of each style (see Supplementary Figure 11). Correct recognition of lullabies was associated with lower CPP, HNR35 and shimmer values, and higher jitter values. For operatic singing, higher recognition was associated with higher CPP, $f_o$max-min (indicating vibrato) and shimmer values, and lower HNR35, tempo and pitch inaccuracy. For pop, correct recognition was associated with higher CPP, pitch inaccuracy, tempo and shimmer values. Note that we included the Energy measure for transparency, but its role here is difficult to interpret.

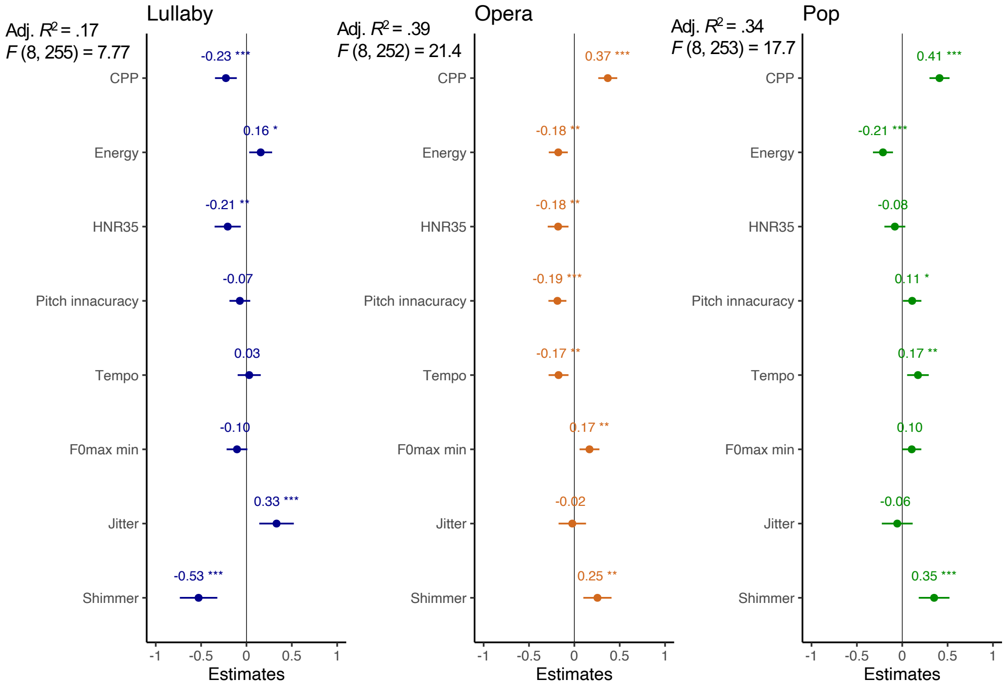

Supplementary Figure 11: Plots of coefficients of regression models predicting the proportion of correct responses in the **main experiment** from acoustic features. Coefficients are standardized by one standard deviation and displayed above their 95% confidence intervals.

The control experiment suggested loudness was important, but not essential to style recognition. We repeated the above analysis with data from the control experiment (see Supplementary Figure 12) and found a roughly similar pattern.

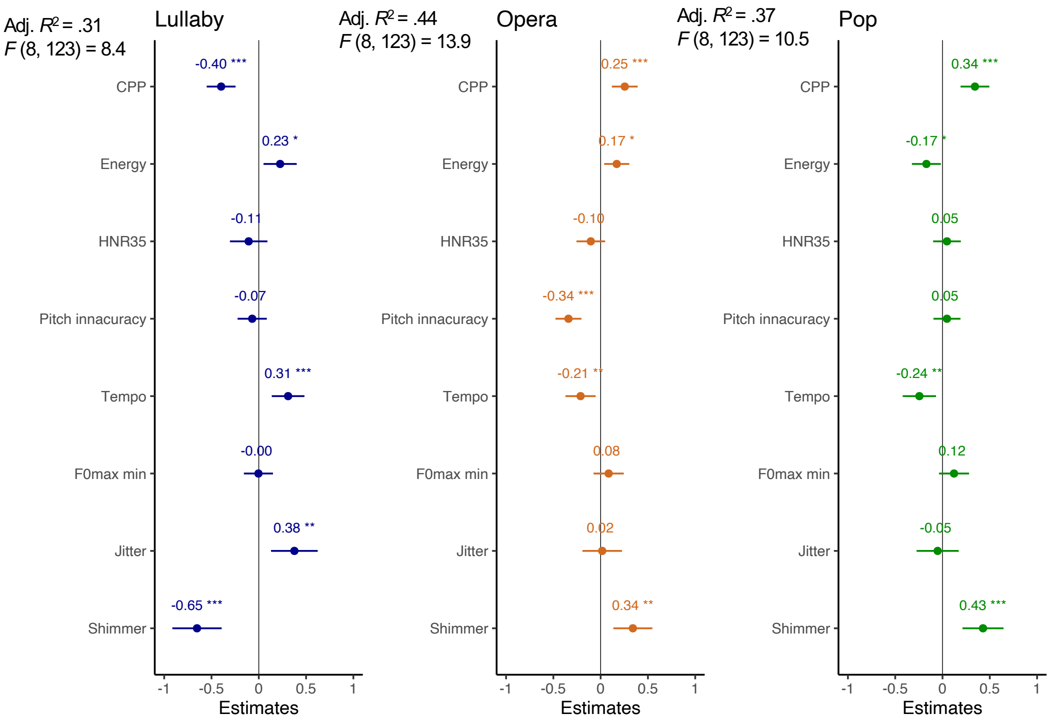

Supplementary Figure 12: Plots of coefficients of regression models predicting the proportion of correct responses in the **control experiment** from acoustic features. Coefficients are standardized by one standard deviation and displayed above their 95% confidence intervals.

It's also important to keep in mind that this exploratory analysis only predicts acoustic features related to correct stimulus recognition, that is, it corresponds only to participants' "correct" perception, but ignores trials where their answers were wrong.

Note a similar pattern of results was found when modeling non-aggregated accuracy data (that is, accuracy from individual trials, coded as 0 = incorrect, 1= correct) and predicting accuracy of recognition in individual trials from acoustics. Please find accompanying .Rmd files (or corresponding .html renderings) for these generalized linear mixed models at https://osf.io/6eyuc/
